# Plasma Gelsolin Confers Chemoresistance in Ovarian Cancer by Resetting the Relative Abundance and Function of Macrophage Subtypes

**DOI:** 10.3390/cancers14041039

**Published:** 2022-02-18

**Authors:** Meshach Asare-Werehene, Hideaki Tsuyoshi, Huilin Zhang, Reza Salehi, Chia-Yu Chang, Euridice Carmona, Clifford L. Librach, Anne-Marie Mes-Masson, Chia-Ching Chang, Dylan Burger, Yoshio Yoshida, Benjamin K. Tsang

**Affiliations:** 1 Department of Obstetrics & Gynecology, Faculty of Medicine & Interdisciplinary School of Health Sciences, Faculty of Health Sciences, University of Ottawa, Ottawa, ON K1H 8L1, Canada; masare@toh.ca (M.A.-W.); rsalehikarashk@ohri.ca (R.S.); 2 Department of Cellular and Molecular Medicine & The Centre for Infection, Immunity and Inflammation (CI3), Faculty of Medicine, University of Ottawa, Ottawa, ON K1H 8M5, Canada; dburger@uottawa.ca; 3 Chronic Disease Program, Ottawa Hospital Research Institute, Ottawa, ON K1H 8L6, Canada; zhl068@njmu.edu.cn; 4 Department of Obstetrics and Gynecology, University of Fukui, Fukui 910-8507, Japan; gth@u-fukui.ac.jp; 5 Department of Obstetrics and Gynecology, Women’s Hospital of Nanjing Medical University, Nanjing Maternity and Child Health Care Hospital, Nanjing 210004, China; 6 CReATe Fertility Centre, 790 Bay Street, Suite 1100, Toronto, ON M5G 1N8, Canada; clifford.librach@utoronto.ca; 7 Department of Biological Science and Technology, Department of Electrophysics and Center for Intelligent Drug Systems and Smart Bio-devices (IDS2B), National Yang Ming Chiao Tung University, Hsinchu 30068, Taiwan; changcy.ac95g@g2.nctu.edu.tw (C.-Y.C.); ccchang01@faculty.nctu.edu.tw (C.-C.C.); 8 Institute of Physics, Academia Sinica, Nankang, Taipei 11529, Taiwan; 9 Centre de Recherche du Centre Hospitalier de l’Université de Montréal and Institut du Cancer de Montréal, Montreal, QC H2X 0A9, Canada; euridice.carmona.chum@ssss.gouv.qc.ca (E.C.); anne-marie.mes-masson@umontreal.ca (A.-M.M.-M.); 10 Departments of Obstetrics & Gynecology and Physiology, Institute of Medical Sciences, University of Toronto, Toronto, ON M5S 1A1, Canada

**Keywords:** tumor-associated macrophages (TAMs), plasma gelsolin (pGSN), ovarian cancer (OVCA), small extracellular vesicles (sEV), chemoresistance

## Abstract

**Simple Summary:**

Ovarian cancer is one of the deadliest female cancers with very poor survival, primarily due to late diagnosis, recurrence and chemoresistance. Although the over-expression of plasma gelsolin (pGSN) protects ovarian cancer cells from chemotherapy-induced death, its immunological role in the tumor microenvironment is less explored. Here, we demonstrate that pGSN over-expression downregulates the anti-tumor functions of M1 macrophages, an effect that contributes to chemoresistance and poor patient survival. This study demonstrates the novel inhibitory role of pGSN on tumor-infiltrated M1 macrophages and also offers new insights in maximizing the effectiveness of immunotherapy for ovarian cancer patients.

**Abstract:**

Ovarian cancer (OVCA) is the most lethal gynaecological cancer with a 5-year survival rate less than 50%. Despite new therapeutic strategies, such as immune checkpoint blockers (ICBs), tumor recurrence and drug resistance remain key obstacles in achieving long-term therapeutic success. Therefore, there is an urgent need to understand the cellular mechanisms of immune dysregulation in chemoresistant OVCA in order to harness the host’s immune system to improve survival. The over-expression of plasma gelsolin (pGSN) mRNA is associated with a poorer prognosis in OVCA patients; however, its immuno-modulatory role has not been elucidated. In this study, for the first time, we report pGSN as an inhibitor of M1 macrophage anti-tumor functions in OVCA chemoresistance. Increased epithelial pGSN expression was associated with the loss of chemoresponsiveness and poor survival. While patients with increased M1 macrophage infiltration exhibited better survival due to nitric-oxide-induced ROS accumulation in OVCA cells, cohorts with poor survival had a higher infiltration of M2 macrophages. Interestingly, increased epithelial pGSN expression was significantly associated with the reduced survival benefits of infiltrated M1 macrophages, through apoptosis via increased caspase-3 activation and reduced production of iNOS and TNFα. Additionally, epithelial pGSN expression was an independent prognostic marker in predicting progression-free survival. These findings support our hypothesis that pGSN is a modulator of inflammation and confers chemoresistance in OVCA, in part by resetting the relative abundance and function of macrophage subtypes in the ovarian tumor microenvironment. Our findings raise the possibility that pGSN may be a potential therapeutic target for immune-mediated chemoresistance in OVCA.

## 1. Introduction

Ovarian cancer (OVCA) is ranked the fifth most commonly diagnosed female cancer; however, it is the most fatal amongst all gynecologic cancers [1,2,3]. This is due primarily to late diagnosis, recurrence and chemoresistance, resulting in no significant improvement in the 5-year survival rate in the last decade [1,2,3]. Although cancer immunotherapy has improved the survival of patients with melanoma, colorectal cancer and blood cancers, it has not achieved great therapeutic success in OVCA patients [4,5,6,7], regardless of the ethnic backgrounds of the patients.

Interestingly, Asians with metastatic solid cancers other than OVCA receiving immunotherapy have significantly improved survival benefit compared with non-Asians, although the reason for the difference has not been investigated [8]. Plasma gelsolin has been shown to be a key factor in suppressing immune responses as well as chemosensitivity in OVCA patients from Western countries [9,10]. As to whether plasma gelsolin has a similar immuno-suppressive role in Asian patients is yet to be investigated. There is therefore an urgent need to better understand the molecular and cellular basis of these poor outcomes in patients with diverse ethnic backgrounds and to investigate novel therapeutic targets to maximize survival in OVCA patients. In this era of personalized therapy, the value of the targets could help to stratify different levels of platinum and immune sensitivities to further refine treatment recommendations and to be used as prognostic markers for the counseling and follow up of these patients.

Tumor-associated macrophages (TAMs) possess immuno-modulatory functions and, depending on their phenotypic state, may promote either tumor progression or regression [11,12]. TAMs are conventionally classified as M1 or M2 depending on the cytokine components of their environment. M1 macrophages are pro-inflammatory and have the ability to phagocytose, which are functions associated with an anti-tumor effect, enhanced treatment response and overall improvement in patient survival [13,14]. The higher tumor infiltration of M1 macrophages is associated with improved patient survival, and this correlation has been shown in a plethora of neoplastic diseases such as ovarian, colorectal, breast, gastric, lung and head-and-neck cancers [11,12,15,16,17]. TAMs exhibiting M2 phenotypes are classically anti-inflammatory and are involved in wound repair and pro-tumorigenesis actions. The immuno-suppressive functions of M2 macrophages have been implicated in breast, ovarian, prostate, lung and brain cancers. The ratio of M1-to-M2 provides prognostic significance in OVCA patients and has been shown to correlate with tumor recurrence and chemoresistance [13,14,18]. However, the mechanism involved in regulating TAMs infiltration and anti-tumor functions in the context of chemoresistance have not been well studied.

Plasma gelsolin (pGSN), also known as secreted gelsolin (sGSN), is a multi-functional actin binding protein and the secreted isoform of the gelsolin (GSN) gene [19,20]. Just like total GSN, pGSN has been implicated in many conditions such as bacterial infection, sepsis, arthritis and cancer [19,21]. In our previous studies, we have shown that pGSN is transported by small extracellular vesicles (sEVs)—often referred to as exosomes which measure approximately 30–150 nm in size [9,22]—which can auto-regulate their own gene expression and, in a paracrine manner, transform chemosensitive cells to acquire a chemoresistant phenotype [9]. Thus, sEV-pGSN is a determinant of OVCA chemoresistance. OVCA patients whose tumor tissues show lower expression of pGSN mRNA have prolonged progression-free survival compared with those with higher pGSN mRNA expression [9]. In addition to OVCA, pGSN has been implicated in other types of cancer such as breast, prostate and colon [9,15,23,24,25,26,27]. Recently, we have also demonstrated that aside from the tumor expression of pGSN, circulating pGSN is a novel candidate for the early detection of OVCA, as well as the prediction of residual disease [28]. To date, the involvement of pGSN in the regulation of the immune system is still not well understood.

After the emergence of checkpoint blockers that activate T cells, one could have expected OVCA patients would respond well to this new therapeutic. Unfortunately, immunotherapy with checkpoint blockers has shown relatively low success in ovarian cancer [5,7]. It has therefore become important to investigate other immune cells, such as macrophages, to determine if they hold the key to maximizing the effectiveness of immunotherapy. M1 macrophages produce NO, which enhances sensitivity to chemotherapy, resulting in the improved survival of cancer patients [29,30,31,32]. Interestingly, cancer cells could evade immune-surveillance by suppressing the production of NO through an unknown mechanism [29,30,31]. Nitric oxide (NO) is a lipophilic free radical that is significantly produced by inducible nitric oxide synthase (iNOS) by transforming L-arginine to L-citrulline [29]. NO is implicated in OVCA transformation, metastasis and progression, although its exact role in chemoresistance is less understood [29,30,31]. In OVCA chemoresistance, increased pGSN expression [9] and lower NO production [29,30,31] have been reported in separate studies; however, it is yet to be determined if these two phenomena are related. To date, the involvement of pGSN in the regulation of the immune system is still not well understood. As to whether pGSN over-expression regulates NO synthesis and suppresses immune cell function and contributes to chemoresistance, this is yet to be examined.

In this study, we investigated the immuno-suppressive role of pGSN in the tumor microenvironment (TME) of Japanese OVCA patients. Specifically, increased tumor pGSN expression attracts M1 macrophages and suppresses their viability and function without affecting M2 macrophages, an action that regulates the inflammatory environment by decreasing the M1/M2 ratio. These results support the notion that these events contribute to chemoresistance and shortened patient survival.

## 2. Materials and Methods

Ethics Statement: All patients provided a written informed consent. The study was performed in accordance with the appropriate guidelines approved by the institutional review board of the University of Fukui Hospital (IRB approval number; 20180140) and the Ottawa Health Science Network Research Ethics Board (IRB approval number; OHSN-REB 1999540-01H).

Tissue Samples: The study included tumor tissues with various histologic subtypes collected from 94 OVCA patients receiving treatment from 2007 to 2018 at the University of Fukui Hospital, Fukui, Japan. Patients were diagnosed, and clinicopathological parameters and follow-up data were gathered. Details of the patient population and demographics as well as inclusion and exclusion criteria are outlined in Supplementary Table S1. Ovarian serous cystadenocarcinoma datasets publicly available on cbioportal (https://www.cbioportal.org/, accessed on 5 August 2020) were interrogated: TCGA, Firehouse legacy. The mRNA expression patterns of pGSN (GSN) and TAMs-associated markers were analyzed in each patient and presented as a heat map. The association between pGSN and M1-macrophage-associated genes was analyzed using Pearson correlation tests. Significant correlations were inferred as *p* ≤ 0.05.

Immunohistochemistry (IHC): Cryostat sections (2.5 μm) were obtained from 94 formalin-fixed, paraffin-embedded ovarian cancer tissues. Tissue samples were immuno-stained (overnight) with primary antibodies to pGSN (×1000), CD68 (total macrophage M0 marker; ×50), HLA-DR (M1 marker; ×1000) and CD163 (M2 marker; ×400). After primary incubation, sections were washed 3× in PBS for 5 min and then incubated (30 min, RT) with secondary antibody (MAX-PO (MULTI)). Immunoreaction was monitored. Tissues were then counterstained (nuclei staining) with Mayer’s Hematoxylin solution and mounted. Tissue sections treated with no primary antibodies were used as negative controls. Staining intensity and the distribution of pGSN were evaluated by two blinded, independent observers using a semi-quantitative method (IRS-score), as described previously [33]. The IRS-score was calculated as follows: IRS = SI × PP, where SI is the optical stain intensity graded as 0 = no staining, 1 = weakly stained, 2 = moderately stained and 3 = strongly stained. PP is the degree of positively stained cells defined as 0 = no staining, 1 ≤ 10%, 2 = 11–50%, 3 = 51–80%, and 4 ≥ 81%). Macrophage density was also quantified from 10 representative high-power fields (HPFs) with ×400 magnification by two blinded independent observers. The macrophages were manually counted in both the epithelial and stromal regions, and the sum was calculated as total macrophages in the tumor microenvironment. Tissue sections were observed using Olympus Bx50F-3 (Olympus Optical Co., Tokyo, Japan) and scanned with FlexScan S2000. Details of antibodies are outlined in Supplementary Table S2.

Reagents: Cis-diaminedichloroplatinum (CDDP), phenylmethylsulfonyl fluoride (PMSF), aprotinin, dimethyl sulfoxide (DMSO), sodium orthovanadate (Na_3_VO_4_) and Hoechst 33258 were supplied by Millipore Sigma (St. Louis, MO, USA). pGSN siRNA1 and 2 and scrambled sequence siRNA (control) were purchased from Integrated DNA Technology (Coralville, IA, USA) and Dharmacon (Lafayette, CO, USA), respectively. Recombinant human plasma gelsolin (rh-pGSN) was purchased from Cytoskeleton, Inc, USA and donated by Dr. Chia-Ching Chang. pGSN cDNA and 3.1A vector plasmids were produced in the lab of Dr. Dar-Bin Shieh, National Cheng Kung University Hospital, Taiwan. See Supplementary Table S2 for details on antibodies and reagents.

Cell Lines: THP-1 monocytes were purchased from ATCC (Manassas, VA, USA). HGS cell lines [34,35,36] ((chemosensitive; OV2295, TOV3041G) and chemoresistant; OV90) were kindly provided by Dr. Anne-Marie Mes-Masson (Centre de recherche du Centre hospitalier de l’Université de Montréal (CRCHUM), Montreal, QC, Canada) and were cultured and maintained in OSE medium (Wisent Inc., St-Bruno, QC, Canada; catalog number: 316-030-CL) supplemented with 10% FBS (Millipore Sigma; St. Louis, MO, USA), 250 µg/mL amphotericin B (Wisent Inc., St-Bruno, QC, Canada; catalog number: 450-105-QL) and 50 mg/mL gentamicin (Wisent Inc., St-Bruno, QC, Canada; catalog number: 450-135-XL). Endometrioid cell lines (A2780, chemosensitive and A2870cp, chemoresistant) were generously donated by Dr. Barbara Vanderhyden (Ottawa Hospital Research Institute, Ottawa, ON, Canada) and were cultured and maintained in Gibco RPMI 1640 (Life Technologies, Grand Island, NY, USA; catalog number: 31800-022) or Dulbecco’s Modified Eagle Medium (Gibco DMEM/F12; Life Technologies, Grand Island, NY, USA; catalog numbers 10565-018/10313-021). Cell lines were authenticated, frequently tested for Mycoplasma contamination using a PlasmoTestTM Mycoplasma Detection kit (Invivogen; catalog number: rep-pt1) and continuously checked for morphological changes as well as growth rate for any batch-to-batch change. RPMI 1640 and DMEM/F12 media were supplemented with 10% FBS (Millipore Sigma; St. Louis, MO, USA), 50 U/mL penicillin, 50 U/mL streptomycin and 2 mmol/L l-glutamine (Gibco Life Technologies, Grand Island, NY, USA). Details on histologic subtypes and genetic alterations of cell lines used are described in Supplementary Table S3. All experiments were carried out in serum-free media.

Gene Interference: Cells were transfected (50 nM, 24 h) with siRNAs (scrambled sequence as controls) using lipofectamine 2000 and harvested for analysis. Two different siRNAs were used for each target to exclude off-target effects. Successful knock-down was confirmed by Western blotting, as previously described [9]. (See Supplementary Table S2 for details on antibodies).

Transient Transfection: Chemosensitive OVCA cells were transfected with pGSN cDNA (2 µg, 24 h) plasmid (empty vector as controls) using lipofectamine 2000 and harvested for further analysis. Successful over-expression was confirmed by Western blotting, as previously described [9,37,38,39].

Extracellular Vesicle Isolation and Characterization: Serum-free conditioned media from cultured cells were used for extracellular vesicle isolation and characterization, as described previously [9]. Total EV concentration was determined by BCA Protein Assay Kit (Thermo Fisher Scientific, Ottawa, ON, Canada). When fresh sEVs (40 µg/400,000 cells) were not required, they were suspended in PBS and stored at −80 °C for subsequent analysis.

Nanoparticle Tracking Analysis (NTA): EVs in PBS were analyzed, using the ZetaView PMX110 Multiple Parameter Particle Tracking Analyzer (Particle Metrix, Meerbusch, Germany) in size mode using ZetaView software version 8.02.28, as previously described [9]. EVs were captured at 11 camera positions at 21 °C and particle size, and concentrations were evaluated.

Protein Extraction and Western blot Analysis: The Western blotting (WB) procedure for proteins were carried out as described previously [9,37,38,39,40]. After protein transfer, membranes were incubated with primary antibodies (1:1000) in 5% (wt/vol) blotto and subsequently treated with the appropriate horseradish peroxidase (HRP)-conjugated secondary antibody (1:2000) in 5% (wt/vol) blotto. Details on antibodies used are described in Supplementary Table S2. Peroxidase activity was visualized using a Chemiluminescent Kit (Thermo Scientific, Rockford, IL, USA). Image J was used to densitometrically measure the signal intensity generated on the film.

ELISA: Concentrations of cytokines were measured using a Multi-Analyte ELISArray Kit (Qiagen, Germantown, MD, USA) in 100 µL of cell-free conditioned media from macrophages after treatment, as previously determined [10]. All ELISA measurements were carried out according to the manufacturer’s instructions. Optical densities (ODs) were determined using a microtiter plate reader at 450 nm and compared to a standard curve. The blank was subtracted from the triplicate readings for each standard and test sample.

THP-1 Monocyte Differentiation and Macrophage Polarization: THP-1 monocytes were treated with PMA (150 nM; 24 h) with RPMI. Treatment was removed, and cells were left to grow in PMA-free RPMI for another 24 h. M0 macrophages were polarized to M1 macrophages with IFNγ (20 ng/mL) + LPS (10 pg/mL) treatment for 24 h and to M2 macrophages with IL-4 (20 ng/mL) + IL-13 (20 ng/mL) for 72 h.

Macrophage differentiation and flow cytometry: Treated cells were collected (2 × 10^5^) and transferred into a round-bottom plate and centrifuged at 200× *g* for 5 min. Cells were then suspended and washed in 200 µL of staining buffer (PBS + 1%FBS). The Fc regions were blocked using 50 µL of staining buffer + Fc block (0.5 µL per 0.1 M cells) and incubated for 10 min at RT. Cells were stained directly with antibody (2 µL of antibody per 50 µL) without removing the Fc block and incubated for 30 min at RT in the dark. In total, 150 µL of staining buffer was added to the cells, centrifuged (200× *g*; 5 min) and washed 2× with 200 µL of staining buffer. Cells were then analyzed using flow cytometry. Unstained and isotype controls for specific primary antibodies were used. For intracellular staining, cells were fixed with 2% paraformaldehyde (30 min; 4 °C), washed 2× in staining buffer and permeabilized in 100 µL of perm buffer (PBS + 1% FBS + 0.01% Sodium Azide + 0.2% Saponin) for 30 min at 4 °C before Fc blocking. Details of the antibodies used are described in Supplementary Table S2.

Macrophage Chemotaxis Assay: M1 and M2 macrophages (2.5 × 10^5^) in 300 µL of RPMI-1640 media were seeded in the upper chambers of 24-Transwell plates (8.0 μm pore size), whereas the lower chambers were seeded with 4 × 10^5^ OVCA cells in 750 µL of RMPI-1640 media or different treatments for 24–48 h. The media was removed, and cells (upper chamber) were washed 2× in PBS before being fixed in 3.7% formaldehyde in PBS for 2 min at room temperature (RT). Cells were then washed 2× in PBS and permeabilized with 100% methanol for 20 min at RT. Cells were then stained with Hoechst stain (15 min; RT in the dark) and washed 2×in PBS. The non-migrated cells were scraped off with cotton wool swabs, and migrated cells were counted under the microscope. The chemotaxis index was calculated as the ratio of migrated cells from treatment groups to control migrated cells.

Assessment of Cell Proliferation and Apoptosis: Apoptosis and cell proliferation were assessed morphologically using Hoechst 33258 nuclear stain [9,26,38] and colorimetrically with CCK-8 assay [10], respectively. A “blinded” counting approach was used to prevent experimental bias with the Hoechst 33258 nuclear staining.

Annexin V flow cytometry: Treated cells were washed in cold 1X PBS before being suspended in binding buffer at a concentration of 1 × 10^6^ cells/mL. Cells were stained with annexin V-FITC solution (1 µL of annexin V stain in 100 µL of binding solution) and incubated for 15 min. Cells were washed in 1X binding buffer and analyzed immediately via flow cytometry. Details of the annexin V-FITC are described in Supplementary Table S2.

Caspase-3 Detection Assay: Caspase-3 activation was detected in treated cells by washing the cells in PBS and staining with 100 µL of working solution as recommended by the manufacturer (Fisher Scientific, Ottawa, Canada; Cat #: C10723). Cells were then fixed with 3.7% formaldehyde (15 min) and counterstained with Dapi (3 min). Cells on the slides were mounted and examined microscopically. The percentage of caspase-3 positive M1 macrophages of the total cells per field was determined.

iNOS Detection Assay: Treated cells were washed with assay buffer and stained with the working solution (1 h, 37 °C) (1:200), as recommended by the manufacturer (Abcam, Toronto, ON, Canada; Cat. #: ab211085). Cells were then analyzed in a microplate reader (Ex/Em = 485 nm/530 nm).

Reactive Oxygen Species (ROS) Detection: Treated cells were washed 3× with PBS and treated with 2′,7′-Dichlorofluorescein diacetate (5 µM; 30 min) at 37 °C. Cells were then washed 3× in PBS, and their fluorescent images were taken using ZOE Fluorescent Cell Imager (Bio-Rad, Mississauga, Canada)

Intracellular Glutathione (GSH) Detection: Here, 1.6 × 10^6^ cells were seeded and treated in DMEM media without serum, and cell lysates (100 µL) were prepared. Intracellular GSH was colorimentrically assessed at 450 nm, using a GSH detection assay kit (ab239727) and a microtiter plate reader as previously analyzed [10]. Concentrations of intracellular GSH were presented as µM/mg protein.

Statistical Analyses: Statistical analyses were performed using the SPSS software version 25 (SPSS Inc., Chicago, IL, USA) and PRISM software version 8.0 (Graphpad, San Diego, CA, USA). The statistical analyses were performed using a independent sample *t*-test, one- or two-way ANOVA and Bonferroni post hoc tests to determine the differences between multiple experimental groups. Two-sided *p* ≤ 0.05 was inferred as statistically significant. The relationship of variables to other clinicopathologic correlates was examined using the Fisher exact test, *t*-test and Kruskal–Wallis Test, as appropriate. Survival curves (PFS and OS) were plotted with Kaplan–Meier and *p*-values calculated using the log-rank test. Univariate and multivariate Cox proportional hazard models were used to assess the hazard ratio (HR) for pGSN, M0, M1 and M2 macrophages, stage, RD and age as well as corresponding 95% confidence intervals (CIs).

## 3. Results

### 3.1. Patients’ Characteristics

Detailed histologic subtype (high-grade serous, HGS; low-grade serous, LGS; endometrioid; mucinous; clear cell and carcinosarcoma) descriptions of OVCA patients (N = 94) are provided in Supplementary Table S1. A certified Gynecologic Oncology team performed tumor staging and pathology. Patients recruited in the study did not receive neoadjuvant chemotherapy prior to sample collection at surgery. The age range of patients was 29–87 years (median age; 56 years) with FIGO stages classified as 1 (N = 55), 2 (N = 9), 3 (N = 23) and 4 (N = 7) (Supplementary Table S1). Seventy-nine (79) patients received complete/optimal cytoreduction compared with fifteen patients who had suboptimal cytoreduction. The median progression-free survival (PFS) and overall survival (OS) were 33.5 and 56.2 months, respectively (Supplementary Table S1).

### 3.2. Increased Epithelial pGSN Expression Is Associated with Suppressed Survival Benefits of Infiltrated M1 Macrophages in OVCA Patients

**Figure 1:**
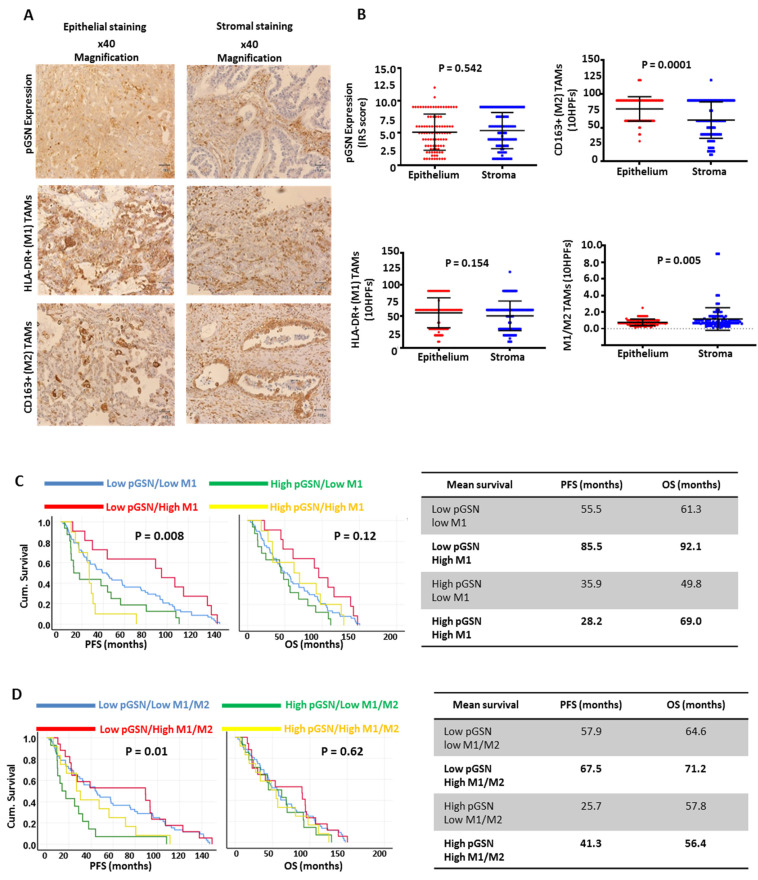
Increased epithelial pGSN expression is associated with suppressed survival benefits of infiltrated M1 macrophages in OVCA patients. (**A**) Ninety-four OVCA tissues were immunostained with anti-pGSN, anti-HLA-DR (M1 macrophage marker) and anti-CD163 (M2 macrophage marker) antibodies in the epithelial and stroma compartments. (**B**) pGSN expression and tissue infiltrated macrophages (M1, M2 and M1/M2) were quantified, compared between epithelial (*n* = 94) and stromal (*n* = 94) regions and presented as scatter plots (mean ± SD). *p*-values were calculated by independent sample t-test. Scale bar is 50 μm. pGSN expression (cut-off = 6) was assessed together with infiltrated (**C**) M1 (cut-off = 60) and (**D**) M1/M2 macrophages (cut-off = 1) in the epithelial region and then correlated with PFS and OS. Kaplan–Meier survival curves with cut-off values and log rank test were used to compare the survival distributions between the groups.

Chemoresistant OVCA cells express and secrete higher levels of pGSN compared with their sensitive counterparts [9]. Thus, we examined the clinical relevance of pGSN as well as infiltrated tumor-associated macrophages (TAMs) in OVCA tissues. Ovarian tissue sections (2.5 μm) were collected from 94 OVCA patients and immunohistochemically stained with anti-pGSN, anti-CD68 (M0 macrophage marker), anti-HLA-DR (M1 macrophage marker) and anti-CD163 (M2 macrophage marker). Primary antibodies were omitted in tissues used as controls. Figure 1A and Supplementary Figure S1A show pGSN expressions as well as the quantity of TAMs (M0 (CD68), M1 (HLA-DR) and M2 (CD163)) in the epithelial (cancer islet) and stromal regions. pGSN expression (cut-off = 6) and TAMs (cut-off = 60) quantity in OVCA tissue compartments with respect to the number of patients is described in Supplementary Table S4. pGSN expression was not significantly different between epithelial and stromal regions (Figure 1B). CD68+ macrophage (M0) infiltration in the stroma was higher compared with that of the epithelial region (Supplementary Figure S1B). However, there was no significant difference in M1 macrophage quantity between epithelial and stromal regions, although M2 infiltration was higher in the epithelial region (Figure 1B). The M1/M2 ratio (cut-off = 1) was significantly higher in the stroma compared with the epithelial compartment (Figure 1B). This could result in a suppressed inflammatory environment in the tumor islet, an action that promotes tumor progression and chemoresistance.

Patients were grouped into sub-categories based on their level of pGSN expression and TAM infiltration (low or high), and their survival benefits were determined. A significant (*p* = 0.008) difference was observed between the groups and PFS but not OS (*p* = 0.12) (Figure 1C). Patients with low pGSN–low M1 macrophages, low pGSN–high M1 macrophages, high pGSN–low M1 macrophages and high pGSN-high M1 macrophages had mean PFSs of 55.5, 85.5, 35.9 and 28.2 months, respectively (Figure 1C). A significant difference (*p* = 0.01) was also observed with PFS but not OS when stratified by M2 macrophage infiltration (Supplementary Figure S1C). Patients with low pGSN–low M2 macrophages, low pGSN-high M2 macrophages, high pGSN–low M2 macrophages and high pGSN-high M2 macrophages had mean PFSs of 68.1, 54.3, 32.9, 33.0 months, respectively (Supplementary Figure S1C). When stratified by the M1/M2 macrophage ratio, a significant association was observed with PFS (*p* = 0.01) but not with OS (*p* = 0.62). Patients with low pGSN-low M1/M2, low pGSN-high M1/M2, high pGSN-low M1/M2 and high pGSN-high M1/M2 had mean PFSs of 57.9, 67.5, 25.7 and 41.3 months, respectively (Figure 1D). This suggests that a pro-inflammatory environment with reduced pGSN expression is key to prolonging tumor recurrence.

### 3.3. Epithelial pGSN Expression, M1/M2 Density and Survival in OVCA Histologic Subtypes

The patients were stratified according to their histologic subtypes, and their survival impact was determined. No significant difference was observed with both PFS (*p* = 0.894) and OS (*p*=0.878) (Supplementary Figure S2A). The epithelial pGSN expression and M1/M2 ratio in patients grouped into histologic subtypes were quantitated and compared. Epithelial pGSN was highly (cut-off = 6) expressed in patients with HGS (mean ± SD (6.0 ± 2.8)), endometrioid (mean ± SD (6.5 ± 2.6)) and LGS (mean ± SD (6.5 ± 1.7)) compared with clear cell (mean ± SD (3.2 ± 1.9)), mucinous (mean ± SD (4.9 ± 3.1)) and carcinosarcoma (mean ± SD (4.8 ± 2.0)) (Supplementary Figure S2B). M1/M2 ratio (cut-off = 1) in the epithelium was higher in patients with HGS (mean ± SD (1.0 ± 0.4)), mucinous (mean ± SD (1.0 ± 0.7)), endometrioid (mean ± SD (1.1 ± 0.6)) and LGS (mean ± SD (1.0 ± 0.3)) compared with patients with clear cell (mean ± SD (0.6 ± 0.5)) and carcinosarcoma (mean ± SD (0.5 ± 0.3)) (Supplementary Figure S2C).

### 3.4. Chemoresistance Is Associated with Increased Epithelial pGSN Expression and M2 Infiltration

**Figure 2:**
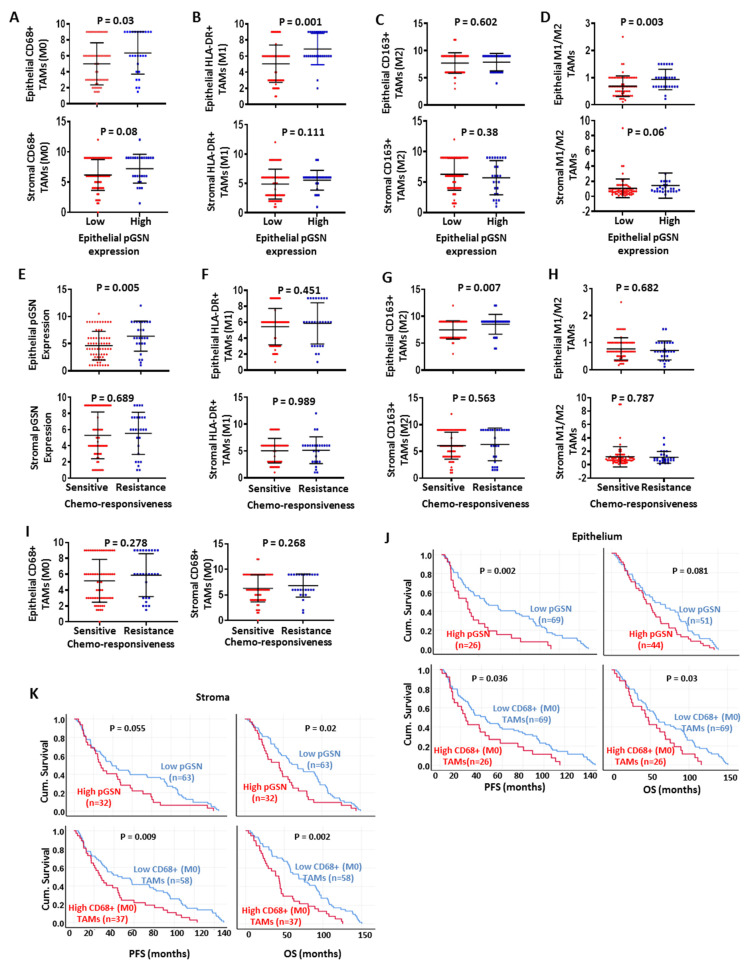
Chemoresistance is associated with increased epithelial pGSN expression and M2 infiltration. Ninety-four OVCA patients were stratified into two groups depending on their level of pGSN expression (low; *n* = 69 vs. high; *n* = 25). (**A**) M0, (**B**) M1, (**C**) M2 and (**D**) M1/M2 densities in the epithelium and stroma were compared and represented as scatter plots (mean ± SD). *p*-values were calculated by independent sample t-test. Patients were also grouped into chemoresistant (PFI ≤ 12 months; *n* = 67) and chemosensitive (PFI > 12 months; *n* = 27) groups. Epithelial and stromal expressions of (**E**) pGSN, (**F**) M1, (**G**) M2, (**H**) M1/M2 and (**I**) M0 were quantitated, compared and represented as scatter plots (mean ± SD). *p*-values were calculated by two sided non-parametric Mann–Whitney test. (**J**) Epithelial and (**K**) stromal pGSN expression (cut-off = 6) and infiltrated M0 (CD68+) macrophages (cut-off = 60) were correlated with PFS and OS. Kaplan–Meier survival curves of categorized pGSN expression (low and high group, cut-off = 6) and M0 density (low and high group, cut-off = 60) and log rank test were used to compare the survival distributions between the groups. *n* = number of patients in each group.

Before determining the relationship between epithelial pGSN and chemoresistance, we assessed TAMs density between patients with high and low epithelial expression of pGSN. We observed that patients with high epithelial pGSN expression had significantly higher epithelial infiltration of CD68+ macrophages (M0; *p* = 0.03), HLA-DR+ M1 macrophages (*p* = 0.001) and M1/M2 ratio (*p* = 0.003) but not CD163+ M2 macrophages (*p* = 0.602) (Figure 2A–D). Interestingly, no significant difference was seen in the stromal compartments regardless of the TAMs phenotype (Figure 2A–D). To demonstrate the association between chemoresistance and epithelial pGSN expression and TAMs, patients were stratified by their progression-free interval (PFI). Patients were stratified as chemoresistant if the PFI was ≤12 months and chemosensitive if the PFI was >12 months. Patients with a PFI ≤ 12 were significantly associated with increased levels of pGSN expression in the epithelial (*p* = 0.005), but not stromal region (*p* = 0.689) (Figure 2E). Chemoresistant and sensitive patients showed no difference in HLA-DR+ M1 macrophage infiltration regardless of the tissue compartment (epithelium; *p* = 0.451, stroma; *p* = 0.989) (Figure 2F). However, chemoresistant patients had significantly higher infiltration of CD163+ M2 macrophages in the epithelial region (*p* = 0.007), but not the stroma (*p* = 0.563) (Figure 2G). The M1/M2 macrophage ratio showed no significant difference between chemosensitive and resistant patients as stratified (epithelium; *p* = 0.682, stroma; *p* = 0.787) (Figure 2H). No significant difference was also observed between CD68+ M0 macrophages and chemoresponsiveness, regardless of the tissue compartment (epithelium; *p* = 0.278, stroma; *p* = 0.268) (Figure 2I). Since the Japanese cohort was predominantly stage 1, the above analyses were only repeated in this stage. Patients with increased pGSN had a higher infiltration of M1 and M1/M2 regardless of the tumor compartment but not M0 and M2 (Supplementary Figure S2D–G). Meanwhile chemoresistance was observed in patients with higher pGSN expression with a propensity to patients with high epithelial M1 infiltration (Supplementary Figure S2H–L). The expression of pGSN and the relative abundance of HLA-DR+ M1 and CD163+ M2 macrophages in the cancer islet are therefore important factors in determining patients’ sensitivity to treatment.

### 3.5. Increased pGSN Expression Is Associated with Poor Patient Survival, Whereas Increased Epithelial M1/M2 Macrophage Density Is Associated with Improved Patient Survival

The clinical relevance of pGSN expression and macrophage infiltration to patient survival was first assessed. Patients with decreased levels of pGSN had prolonged PFS (epithelium; *p* = 0.002, stroma; *p* = 0.055) and OS (epithelial; *p* = 0.081, stroma; *p* = 0.02) regardless of the tissue compartment (Figure 2J,K). We further examined the survival benefits of the various subtypes of infiltrated TAMs. The lower infiltration of CD68+ macrophages (M0) in both epithelial (PFS; *p* = 0.036, OS; *p* = 0.03) and stromal (PFS; *p* = 0.009, OS; *p* = 0.002) compartments was associated with survival benefits to patients (Figure 2J,K). OVCA patients with higher infiltration of HLA-DR+ M1 macrophages had significantly prolonged PFS (epithelium; *p* = 0.038, stroma; *p* = 0.065) and OS (epithelium; *p* = 0.032, stroma; *p* = 0.042) compared with patients with lower HLA-DR+ M1 infiltration (Supplementary Figure S3A). Unlike M1 macrophages, no significant survival difference in PFS and OS was observed with CD163+ M2 macrophage infiltration, regardless of the tissue compartment (Supplementary Figure S3B). Although increased M1/M2 macrophage density was associated with improved PFS (*p* = 0.018) and OS (*p* = 0.007) in the epithelial region, no significant survival difference was observed in the stroma (PFS; *p* = 0.722, OS; *p* = 0.595) (Supplementary Figure S3C). This suggests that the survival of OVCA patients could be impacted by the level of pGSN expression and the ratio of M1/M2 macrophages present in the cancer nest.

We then investigated the association between pGSN and TAMs density in the tumor microenvironment. A significant positive correlation between epithelial pGSN expression and CD68+ (M0) macrophage infiltration was observed in the epithelial region (r = 0.31; *p* = 0.002) but not the stroma (r = 0.16; *p* = 0.13) (Supplementary Figure S4A), although these correlations were weak. There was a weak but positive significant association between epithelial pGSN expression and HLA-DR+ M1 macrophage density in the epithelial (r = 0.41; *p* = 0.0001) and stromal regions (r = 0.25; *p* = 0.005) (Supplementary Figure S4B). Unlike M1 macrophages, no significant correlation was observed with CD163+ M2 macrophage infiltration and pGSN, regardless of the tissue compartment (epithelium; r = 0.12; *p* = 0.25, stroma; r = 0.11; *p* = 0.30) (Supplementary Figure S4C). When the relationship between M1/M2 macrophage ratio and epithelial pGSN expression was assessed, we observed a weak but positive and significant correlation between epithelial pGSN expression and the M1/M2 macrophage ratio, regardless of tissue compartment (epithelial; r = 0.33; *p* = 0.001, stroma; r = 0.28; *p* = 0.005) (Supplementary Figure S4D). The positive correlation between pGSN and M1 macrophage infiltration is consistent with our findings when 594 OVCA tissues from the TCGA dataset (Firehouse Legacy) were interrogated (Supplementary Figure S5A–B), suggesting that a pro-inflammatory ovarian tumor microenvironment may be an important determinant of the pGSN-mediated reduction in OVCA patient survival.

### 3.6. Prognostic Impact of Epithelial pGSN, TAMs and Other Clinicopathologic Parameters

We assessed the prognostic impact of epithelial pGSN, TAMs and other clinicopathological parameters using uni- and multivariate Cox regression analyses, as shown in Table 1 and Table 2, respectively. Median cut-offs were used to predict PFS and OS. In the univariate Cox regression analysis (Table 1), age, stage (FIGO), residual disease (RD) and M2 macrophage (CD163+) showed a significant association with PFS and OS. Epithelial pGSN only showed a significant association with PFS (HR, 1.233; CI, 1.07–1.420; *p* = 0.004) but not OS (HR, 1.054; CI, 0.870–1.276; *p* = 0.591). In the multivariate Cox regression analysis (Table 2), only epithelial pGSN (HR, 1.181; CI, 1.010–1.381; *p* = 0.038), histologic subtype (HR, 0.547; CI, 0.312–0.954; *p* = 0.035) and RD (HR, 0.138; CI, 0.049–0.391; *p* < 0.001) were found to be significant predictors of PFS. With regard to OS, only RD was significantly associated with an increased risk of death. Taken together, these findings suggest that increased tumor pGSN expression plays a key role in ovarian tumor progression and could serve as a marker for suboptimal residual disease and tumor recurrence.

### 3.7. Chemoresistant-Cell-Derived sEV Attenuates M1 Macrophage’s Anti-Tumor Function by Increased Caspase-3-Dependent Apoptosis and Decreased Secretion of iNOS and TNFα

**Figure 3:**
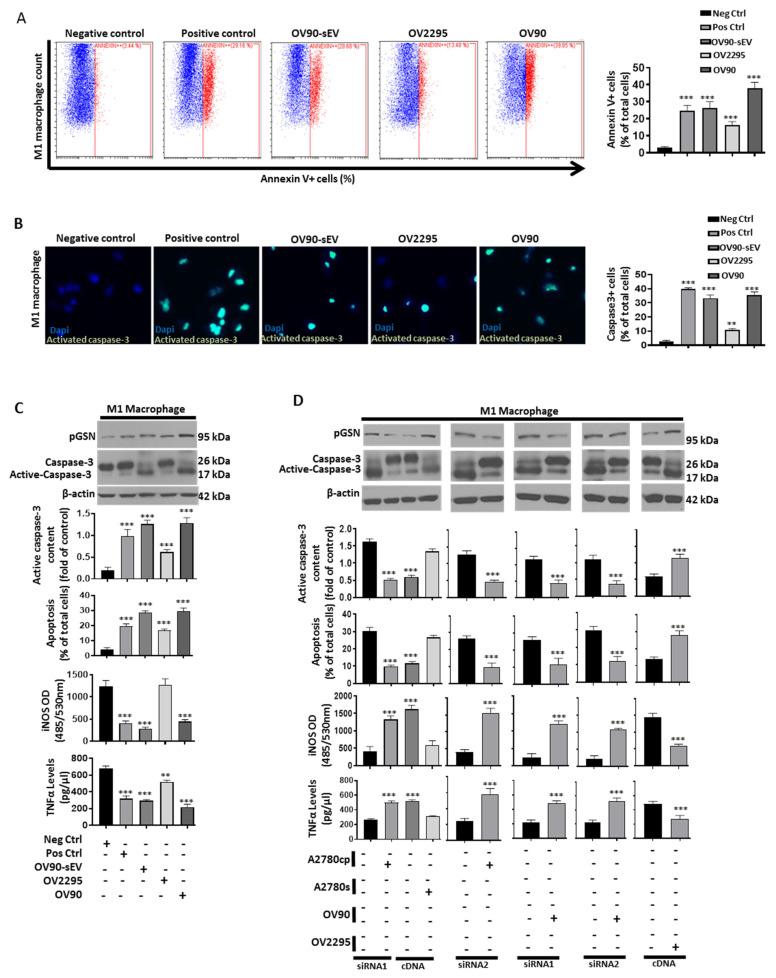
Chemoresistant-cell-derived sEV attenuates the anti-tumor function of M1 macrophages by increased caspase-3-dependent apoptosis and decreased production of iNOS and TNFα. (**A**–**C**) M1 macrophages were co-cultured with serum-free media (negative control; 48 h), etoposide (0.5 µM; 48 h), OV90-derived sEV (40 µg/400,000 cells; 48 h), OV2295 (48 h) and OV90 (48 h). M1 macrophage apoptosis was assessed by annexin V-FITC flow cytometry, caspase-3 activation detection assay, morphologically by Hoechst 33258 DNA staining and Western blot. (**D**) M1 macrophages were co-cultured with OV90/A2780cp (pGSN siRNA1 and 2; 50 nM; 24 h) and OV2295/A2780s (cDNA 2 µg; 24 h). Scramble RNAs and empty vectors were used as control for the knock-down and over-expression, respectively. iNOS abundance (M1 macrophage) and TNFα (M1 macrophage-conditioned media) secretions were determined by fluorometric assay (Ex/Em = 485/530 nm) and ELISA, respectively. Pro-caspase-3, activated caspase-3, pGSN and beta-actin contents were assessed by Western blot (M1 macrophage lysates). Results are expressed as means ± SD from three independent replicate experiments (** *p* < 0.01; *** *p* < 0.001). Scale bar is 100 µm.

In the OVCA tissues, we observed that although increased expression of pGSN is associated with increased M1 macrophage density, patient survival is reduced. We therefore hypothesized that increased pGSN expression draws M1 macrophages into the cancer nest and suppresses their viability, thus leading to a change in the pro-inflammatory environment (M1/M2 ratio) and decreased patient survival. Thus, we further examined, utilizing in vitro techniques, how this phenomenon occurs. Chemoresistant OVCA secretes increased levels of exosomal pGSN that confers cisplatin resistance on otherwise sensitive cells [9] and regulates immune cells. We therefore further hypothesized that exosomal pGSN derived from chemoresistant OVCA cells will induce apoptosis in M1 macrophages, as well as reduce the secretion of iNOS and TNFα. Macrophage differentiation and polarization were confirmed using flow cytometry, ELISA and Western blot (Supplementary Figure S6A–D and Figure S10). M1 macrophages were treated with serum-free RPMI 1640 media (negative control), 0.5 µM etoposide (positive control), sEV (40 µg/400,000 cells) and co-cultured with chemosensitive (HGS, OV2295 and endometrioid, A2780s) and chemoresistant OVCA cells (HGS, OV90; endometrioid, A2780cp) for 48 h (Figure 3, Supplementary Figure S11). The apoptosis of M1 macrophages was assessed using annexin V flow cytometry (Figure 3A) and caspase-3 fluorescence microscopic detection (Figure 3B) as well as Western blotting and Hoechst staining (Figure 3C and Supplementary Figure S7A). Chemoresistant-cell-derived sEVs and chemoresistant OVCA cells significantly induced M1 macrophage apoptosis (assayed morphologically by Hoechst nuclear staining; Figure 3C and Supplementary Figure S7A), compared with chemosensitive cells, which was evidenced by increased annexin v+ cells and increased caspase-3 activation (Figure 3C, Supplementary Figure S7A, Figure S11 and Figure S12). Tumor-infiltrated M1 macrophages secrete anti-tumor factors such as iNOS and TNFα, which induce apoptosis in cancer cells, thereby contributing to improved patient survival [41,42]. Thus, we examined iNOS and TNFα production by M1 macrophages after their co-culture with OVCA cancer cells. iNOS and TNFα secretions by M1 macrophages were significantly decreased when treated with chemoresistant-cell-derived sEVs or co-cultured with chemoresistant OVCA cells compared with chemosensitive cells, regardless of the histologic subtype (Figure 3C and Supplementary Figure S7A, Figure S11 and Figure S12). This suggests that sEVs derived from chemoresistant OVCA cells, regardless of histologic subtype, are capable of suppressing the viability and anti-tumor functions of M1 macrophages.

### 3.8. Increased pGSN Selectively Attracts and Suppresses M1 Macrophage Survival but Not M2 Macrophages

To demonstrate that pGSN is indeed involved in the induction of M1 macrophage apoptosis as observed above, we performed loss- and gain-of-function studies in which pGSN in chemoresistant OVCA cells (OV90 and A2780cp) was knocked down (KD) with two [2] different siRNAs (50 nM; 24 h), as well as over-expressed (OX) in chemosensitive cells, which otherwise express low pGSN (OV2295 and A2780s) using cDNA (2 µg; 24 h) (Figure 3D, Supplementary Figure S11). Scrambled plasmids were used as controls. pGSN KD and OX were confirmed by Western blotting (Supplementary Figure S7B,C and Figure S12). M1 macrophages were then co-cultured with chemoresistant cells pGSN-KD and chemosensitive cells pGSN-OX and their respective control cells for 48 h. Knocking down pGSN from chemoresistant OVCA cells significantly reduced caspase-3 activation and apoptosis in M1 macrophages, a response that was associated with the increased secretion of iNOS and TNFα (Figure 3D, Supplementary Figure S11). The over-expression of pGSN in chemosensitive OVCA cells resulted in significantly increased caspase-3 activation and apoptosis, which was associated with decreased iNOS and TNFα secretion (compared with control cells; Figure 3D). This suggests that pGSN is an inhibitor of M1 macrophage function.

**Figure 4:**
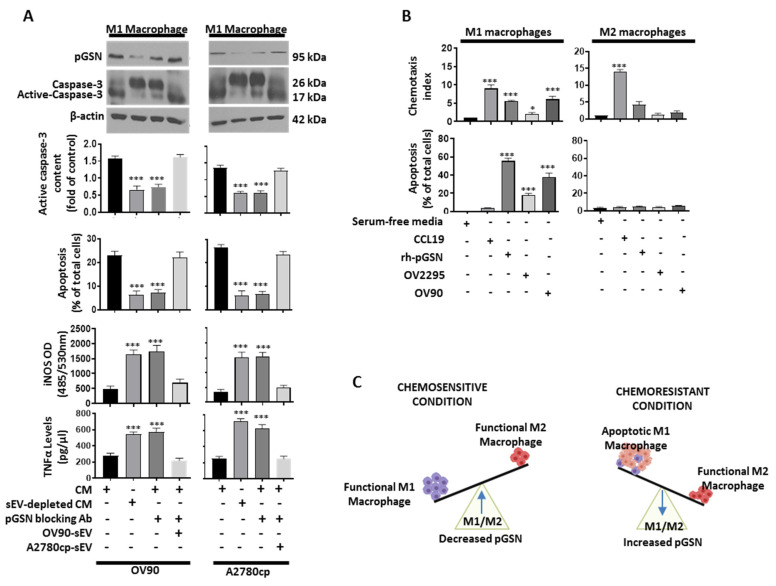
pGSN selectively attract M1 macrophages and suppress their viability without affecting M2 macrophages. (**A**) M1 macrophages were treated with the following: CM + IgG, sEV-depleted CM + IgG (control), CM + pGSN blocking antibody (bAb) and CM + pGSN-bAb + sEVs. CM was derived from chemoresistant cells (OV90 and A2780cp; 3 mL; 48 h). Pro-caspase-3, activated caspase-3, pGSN and beta-actin contents were assessed by Western blotting assay (M1 macrophage lysates) and apoptosis determined morphologically by Hoechst 33258 DNA staining. iNOS (M1 macrophage) and TNFα secretions (M1 macrophages conditioned media) were determined by fluorometric assay (Ex/Em = 485/530 nm) and ELISA, respectively. (**B**) M1 and M2 macrophages were treated with serum-free media (negative control; 3 mL), CCL19 (positive control; 30 nM), rh-pGSN (10 µM) and OVCA cells (co-culture with OV2295 and OV90) for 48 h. Chemotactic assay was used to determine macrophage migration and apoptosis morphologically determined by Hoechst 33258 DNA staining. (**C**) In chemosensitive condition, there is an increase in M1/M2 ratio due to decreased pGSN expression. In chemoresistant condition, M1 macrophages are selectively attracted into the cancer islet and executed without affecting the viability of M2 macrophages. Thus, the M1/M2 ratio is significantly decreased and favors poor survival. Results are expressed as means ± SD from three independent replicate experiments (* *p* < 0.05; *** *p* < 0.001).

To further investigate the role of pGSN in M1 macrophage suppression, M1 macrophages were treated with chemoresistant-cell-derived conditioned media (CM) + IgG, sEV-depleted CM from chemoresistant cells + IgG (control), chemoresistant-cell-derived CM + pGSN blocking antibody (bAb), as well as chemoresistant-cell-derived CM + pGSN-bAb + sEVs (Figure 4A). Increased caspase-3 activation and apoptosis were observed in M1 macrophages when treated with CM + IgG, a response that was attenuated by the presence of pGSN-bAb (Figure 4A and Supplementary Figure S13). Moreover, this blocking effect of the pGSN antibody could be overcome by sEVs (CM + pGSN-bAb + sEVs), a phenomena that was associated with decreased iNOS and TNFα secretion (Figure 4A). M1 macrophage apoptosis was further validated using recombinant human pGSN (10 µM; 24 h) (Supplementary Figure S7D). M1 macrophage apoptosis was increased after rhpGSN treatment, which was marked by increased annexin V+ M1 macrophages (Supplementary Figure S7D). Together, these findings demonstrate that pGSN is the key molecule in the chemoresistant-cell-derived sEVs responsible for suppressing the viability and anti-tumor functions of M1 macrophages.

A co-culture system was established to determine if pGSN has a chemo-attractant effect on M1 and M2 macrophages. The macrophages were seeded in the upper chambers, while the lower chambers either contained serum-free media (negative control; 3 mL), CCL19 (positive control; 30 nM), rh-pGSN (10 µM) or OVCA cells (co-culture with OV2295 and OV90) for 48 h. Chemotaxis assay and Hoechst staining were used to assess macrophage migration and apoptosis, respectively. pGSN selectively attracted and suppressed the viability of M1 macrophages without affecting M2 macrophages, a phenomenon that was also observed with chemoresistant cells (OV90) but not chemosensitive cells (OV2295). This suggests that in patients with high pGSN expression, M1 macrophages are selectively attracted into the cancer islet, and their viability is reduced without affecting the viability of M2 macrophages. Thus, the M1/M2 ratio is significantly decreased and favors poor survival and chemoresistance (Figure 4C).

### 3.9. M1 Macrophage-Derived Nitric Oxide (NO) Sensitizes Chemoresistant OVCA Cells to CDDP-Induced Death via Increased Reactive Oxygen Species (ROS) Production

**Figure 5:**
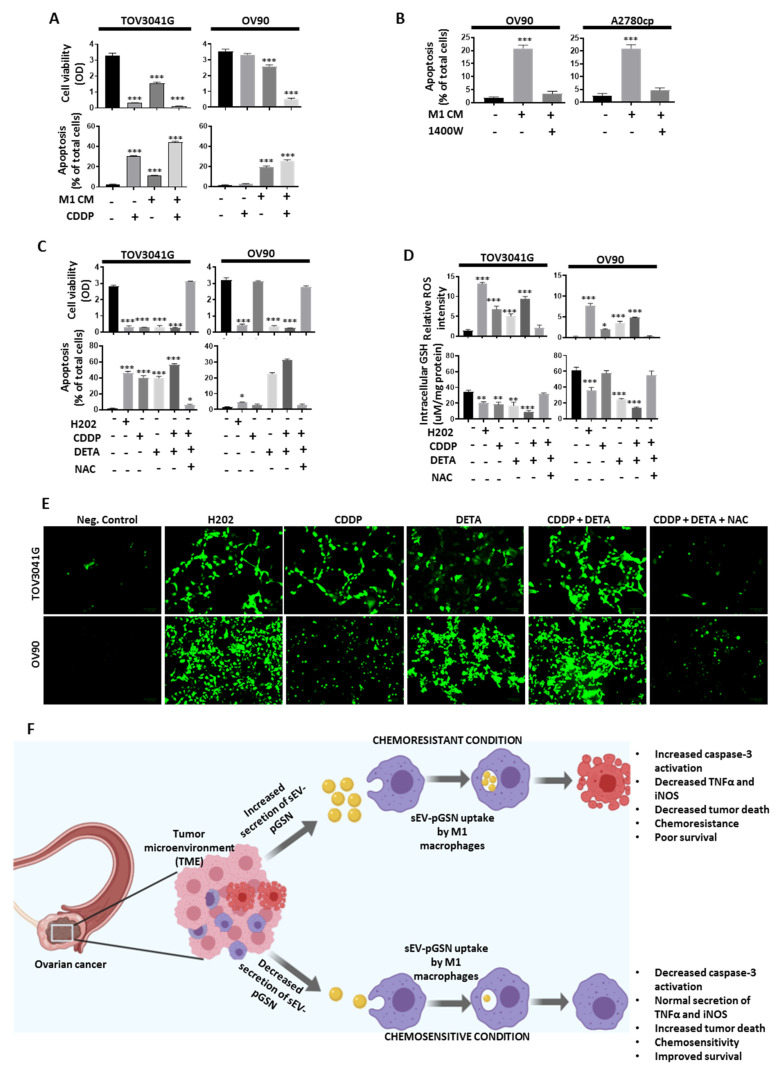
M1 macrophage-derived nitric oxide (NO) sensitizes chemoresistant OVCA cells to CDDP-induced death via increased reactive oxygen species (ROS) production. (**A**) Chemosensitive (TOV3041G) and chemoresistant (OV90) cell lines were treated with M1 macrophage-derived conditioned media (CM, 3 mL; 24 h) and CDDP (10 µM; 24 h). (**B**) M1 macrophages were treated with or without 1400 W (selective inhibitor of iNOS, 100 µM; 24 h). Conditioned media from treated M1 macrophages were collected and used as treatment on chemoresistant OVCA cell lines (OV90 and A2780cp, 3 mL; 24 h). (**C**–**E**) TOV3041G and OV90 cells were treated with H202 (positive control; 2 mM), CDDP (10 µM), DETA NONOate (NO donor, 200 µM), CDDP + DETA NONOate and CDDP (10 µM) + DETA NONOate (200 µM) + N-acetylcysteine (GSH precursor (NAC; 200 µM)) for 6–12 h. Cell viability was measured using CCK-8 assay and apoptosis morphologically determined by Hoechst 33258 DNA staining. Intracellular GSH was measured by colorimetric assay and ROS determined by 2′,7′-Dichlorofluorescein diacetate assay. (**F**) In chemosensitive condition, OVCA cells secrete decreased levels of sEV containing pGSN which have minimal suppression on M1 macrophages. In chemoresistant condition, OVCA cells secrete higher levels of sEV containing pGSN which suppress the anti-tumor functions of M1 macrophages by (i) inducing apoptosis and (ii) decreasing TNFα and iNOS production. Results are expressed as means ± SD from three independent replicate experiments. (* *p* < 0.05; ** *p* < 0.01; *** *p* < 0.001). Scale bar is 100 µm.

NO has been shown to possess anti-tumor properties although its role in chemoresistance is less explored [29]. We thus investigated if NO derived from M1 macrophages could sensitize chemoresistant OVCA cells to CDDP-induced death. Chemosensitive (TOV3041G) and chemoresistant (OV90) cell lines were treated with M1 macrophage-derived conditioned media (CM, 3 mL; 24 h) and CDDP (10 µM; 24 h) (Figure 5A). We demonstrated that M1 macrophage-derived CM did not only suppress OVCA cell survival but also sensitized chemoresistant OVCA cells to CDDP-induced death (Figure 5A). When iNOS was selectively inhibited in M1 macrophages using 1400 W (selective inhibitor of iNOS, 100 µM; 24 h), M1 macrophage-mediated OVCA cell death was attenuated (Figure 5B), suggesting that iNOS was responsible for inducing cell death in the OVCA cell lines. This observation motivated us to further investigate the anti-tumor effects of DETA NONOate (a NO donor) on OVCA cells. TOV3041G, A2780s, A2780cp and OV90 cells were treated with H202 (positive control; 2 mM), CDDP (10 µM), DETA NONOate (NO donor, 200 µM), CDDP + DETA NONOate and CDDP (10 µM) + DETA NONOate (200 µM) + N-acetylcysteine (GSH precursor (NAC; 200 µM)) for 6–12 h (Figure 5C,D and Supplementary Figure S8). We observed that DETA NONOate did not only induce apoptosis in OVCA cells but also sensitized chemoresistant cells to CDDP-induced death, a phenomenon that was significantly attenuated by NAC. Upon further investigation, we demonstrated that NO induces apoptosis by increasing ROS production and decreasing GSH synthesis.

## 4. Discussion

In this present study, for the first time, we demonstrated the role of pGSN as a modulator of inflammation, leading to chemoresistance in OVCA in part by resetting the relative abundance and function of macrophage subtypes in the ovarian tumor microenvironment. Unlike our previous studies which involved patients from Western countries, the cohort used in the current study involved Japanese patients which have an unusual patient distribution in histology (a high number of non-serous cases), age (<average age at diagnosis), tumor stage (~60% stage 1), surgical outcomes (>average complete/optimal residual disease) and treatment regimen (increased treatment response). This diversity could impact patient survival and could explain why these cohorts of patients have relatively higher PFS compared with patients from Western countries. These factors strengthen the point that ethnic differences may play a significant role in ovarian cancer pathology, management and patient survival.

Using this Japanese cohort, we showed that the increased epithelial expression of pGSN is associated with poor survival and chemoresistance. Although there was no significant difference between pGSN expression in the epithelial and stromal regions, the epithelial pGSN expression provided the most clinical relevance. These findings are consistent with our previous studies, where increased pGSN mRNA levels were associated with poor prognosis regardless of treatment regimen or the OVCA histological subtype [9]. This also supports the findings from other studies in which pGSN levels were implicated in head-and-neck, ovarian and prostate cancers [9,23,24,26,27]. Thus, targeting pGSN by way of monoclonal antibodies or small-molecule inhibitors could provide therapeutic advantages to patients with OVCA regardless of their ethnic background. Since pGSN is highly detectable in all histologic subtypes, targeting it could provide therapeutic advantage to patients with HGS and other histologic subtypes.

Harnessing the anti-tumor functions of CD8+ T cells has shown much promise; however, minimal therapeutic success has been achieved [5,42,43]. This has motivated us to investigate other anti-tumor immune cells such as tumor-associated macrophages (TAMs) to determine if they could be re-programmed to kill OVCA cells in the tumor microenvironment. Although there was no significant difference with M1 macrophage infiltration in both histologic compartments, a prolonged survival was observed in patients who had increased infiltration in the cancer islet (epithelial compartment). This is consistent with other findings in breast, colon, lung and head-and-neck cancers, where the infiltration of M1 macrophage but not of M2 was associated with better survival and improved treatment responses [44,45,46,47]. TAMs that exhibit M1 phenotypes are classically pro-inflammatory and have high phagocytic properties. These cells in their functional state also produce increased levels of TNFα and iNOS, which contribute to the killing of tumor cells. Nitric oxide production is associated with decreased cancer progression, metastasis and differentiation [30,31,48]; however, its role in OVCA chemoresistance is less explored. We demonstrated that M1 macrophage-derived, NO-sensitized, chemoresistant OVCA cell to CDDP-induced death by upregulating ROS production and reducing GSH synthesis. These findings are consistent with other reports that have shown iNOS expression in OVCA as a favorable prognostic indicator of disease-related survival [30]. In other reports, nitric oxide donors have been shown to induce cancer cell death by upregulating the expression of RASSF1 and CDKN1A [48], activating caspase 8/3 [49] and regulating anti-apoptotic BCL-2 family members [50]. Whether NO-induced ROS accumulation results in the activation of other pro-apoptotic genes requires further investigation. Re-engineering TAMs to express M1 macrophage phenotypic genes provides anti-tumor effects and has the potential of increasing patients’ survival [46]. Thus, increasing the presence of M1 macrophages in the tumor environment while inhibiting the expression of tumor pGSN could provide survival benefits to patients. This might explain the correlation between increased M1 macrophage infiltration and prolonged survival.

We further investigated the potential relationship between epithelial pGSN expression and TAM infiltration. We observed a significant positive correlation between pGSN expression and the M1/M2 ratio in both the epithelial and stroma compartments. This ratio, indicative of the inflammatory status of the ovarian TME, provides the relative abundance of two cell populations at a time and could be used as a diagnostic index. Interestingly, we observed that, although epithelial pGSN positively correlated with M1 macrophages, the survival impact of the M1 macrophages appeared to be compromised. Specifically, the mean PFS dropped significantly from 85.5 months to 28.2 months. Similar phenomena were seen when the M1/M2 macrophage ratio was used. This was only significant in PFS but not OS. PFS is suggestive of the tumor biology and is a key determining factor for tumor recurrence and chemoresistance. Although this cohort is a mix of different histologies and it is difficult to stratify the biologic effects for each subtype due to the small sample size, the observed findings are intriguing and worth investigating further.

Establishing reliable diagnostic and prognostic factors in cancer, especially OVCA, is key to improving the overall survival of patients. In both uni- and multivariate analysis, epithelial pGSN and residual disease (RD) emerged as independent predictors—amongst all prognostic factors investigated—associated with progression-free survival. Although there was no significant correlation between the epithelial and stromal expression of pGSN, epithelial pGSN expression increased with stage and residual disease (Supplementary Figure S9A–C). Therefore, it is not surprising that epithelial pGSN was found to be an independent prognostic marker, together with RD. This observation is consistent with our previous study where pre-operative circulating pGSN presented as a less-invasive marker for predicting residual disease and indicating stage 1 OVCA [28]. Thus, we were further motivated to investigate the inhibitory role of pGSN in M1 macrophages in vitro.

Chemoresistant OVCA cells secrete increased levels of pGSN that are transported via sEVs [9]. sEVs containing pGSN auto-regulate its gene and confers cisplatin resistance in a paracrine manner to otherwise chemosensitive cells [9]. We therefore hypothesized that exosomal pGSN modulates the pro-inflammatory environment in the ovarian TME in part by down-regulating M1 macrophage viability and function, an effect that lowers the M1/M2 ratios associated with a shortening of patient survival. Upon investigation, we demonstrated that chemoresistant-cell-derived sEVs compared with their sensitive counterparts induced M1 macrophage death via caspase-3 activation, a phenomenon associated with a secondary suppression of iNOS and TNFα production. Silencing the pGSN gene in chemoresistant OVCA cells resulted in the attenuation of M1 macrophage caspase-3 activation, apoptosis and the elevated productions of iNOS and TNFα. These responses were the opposite when pGSN was over-expressed in chemosensitive cells. These observations support our hypothesis and are also consistent with other studies where exosomal pGSN suppresses the anti-tumor functions of immune cells such as CD8+ T cells and natural killer T cells [24,51]. Our findings are also consistent with other studies where recombinant human pGSN reduced inflammatory cytokines such as iNOS, TNFα and IL-6 in LPS-stimulated human keratinocytes [52], RAW264.7 [53], peritoneal macrophages [53] and THP-1 cells [53] as well as carrageenan-induced paw edema in mice [54]. Based on our results, we would predict that combining pGSN inhibitors with other immune checkpoint blockers, such as anti-PD-1 and anti-PDL1, will significantly inhibit tumor growth and unleash the anti-tumor properties of CD8+ T cells, NK cells and M1 macrophages. This will provide a broad anti-tumor approach and could enhance patient survival. To the best of our knowledge, this is the first study to demonstrate the inhibitory role of pGSN on M1 macrophage viability and function in the ovarian TME as well as NO-induced, ROS-mediated OVCA sensitivity. The possibility that pGSN may also alter the phenotype of TAMs is worth considering in future studies.

## 5. Conclusions

In conclusion, for the first time, we demonstrated that increased pGSN modulates the pro-inflammatory environment in the ovarian TME, favoring chemoresistance and poor patient survival. Specifically in the chemosensitive condition, OVCA cells secrete low levels of sEV containing pGSN, rendering them incapable of inhibiting the anti-tumor functions of M1 macrophages (Figure 5F). However, in the chemoresistant condition, OVCA cells secrete increased levels of sEV-containing pGSN that are taken up by M1 macrophages (Figure 5F). Upon uptake, pGSN is released and activates caspase-3, resulting in apoptosis. This is also associated with a secondary response where TNFα and iNOS productions are significantly reduced (Figure 5F). These cumulative effects render infiltrated M1 macrophages non-functional and unable to kill tumor cells. While these findings are novel and are of clinical relevance, we also acknowledge the unusual patient distribution and small population of patients recruited, making it difficult to analyze the biologic effects of each subtype. Thus, future studies that address the above-mentioned concerns together with animal models will provide further validation of our observations and extend the applicability of the current study.

## Figures and Tables

**Table 1:** Univariate Cox regression analysis for PFS and OS.

Univariate						
Variable	PFS			OS		
	HR *	95% CI ^^^	*p*-Value	HR *	95% CI ^^^	*p*-Value
Age (years)						
≤56 vs. >56	1.046	1.012–1.082	0.008	1.049	1.002–1.098	0.041
Stage (FIGO)						
≤2 vs. >2	4.803	2.153–10.717	<0.001	3.682	1.232–11.005	0.020
RD (cm)						
≤1 vs. >1	0.114	0.052–0.251	<0.001	0.117	0.039–0.350	<0.01
pGSN ^epi^						
Low vs. high	1.233	1.07–1.421	0.004	1.054	0.870–1.276	0.591
CD68 ^epi^						
Low vs. high	1.092	0.946–1.26	0.23	1.011	0.832–1.228	0.915
HLA-DR (M1) ^epi^						
Low vs. high	1.035	0.881–1.215	0.676	0.826	0.661–1.033	0.093
CD163 (M2) ^epi^						
Low vs. high	1.328	1.065–1.658	0.012	1.436	1.047–1.971	0.025
M1/M2 ^epi^						
Low vs. high	0.787	0.391–1.584	0.503	0.397	0.138–1.137	0.085
Histologic subtype	0.787	0.549–1.129	0.194	0.806	0.488–1.333	0.401

HR, hazard ratio; PFS, disease free survival; OS, overall survival; CI, confidence interval; RD, residual disease; pGSN, plasma gelsolin; FIGO, International Federation of Gynecology and Obstetrics; vs., versus; ^epi^, epithelial. * Estimated from Cox proportional hazard regression model. ^ Confidence interval of the estimated HR.

**Table 2:** Multivariate Cox regression analysis for PFS and OS.

Multivariate Analysis						
Variable	PFS			OS		
	HR *	95% CI ^^^	*p*-Value	HR *	95% CI ^^^	*p*-Value
Age (years)						
≤56 vs. >56	1.126	0.387–3.279	0.828	1.352	0.331–5.530	0.675
Stage (FIGO)						
≤2 vs. >2	1.090	0.358–3.320	0.880	0.945	0.183–4.874	0.946
RD (cm) ≤1 vs. >1						
	0.103	0.033–0.322	<0.001	0.139	0.032–0.605	0.009
pGSN ^epi^						
Low vs. high	1.300	1.096–1.541	0.003	1.019	0.793–1.309	0.884
CD68 ^epi^						
Low vs. high	0.939	0.764–1.154	0.549	0.943	0.706–1.261	0.694
HLA-DR (M1) ^epi^						
Low vs. high	1.030	0.809–1.312	0.808	0.828	0.552–1.242	0.362
CD163 (M2) ^epi^						
Low vs. high	1.274	0.969–1.674	0.082	1.481	0.910–2.413	0.114
M1/M2 ^epi^						
Low vs. high	0.603	0.198–1.836	0.373	1.173	0.174–7.912	0.870
Histologic subtype	0.547	0.312–0.954	0.035	0.730	0.354–1.503	0.393

HR, hazard ratio; PFS, disease free survival; OS, overall survival; CI, confidence interval; RD, residual disease; pGSN, plasma gelsolin; FIGO, International Federation of Gynecology and Obstetrics; vs., versus; ^epi^, epithelial. * Estimated from Cox proportional hazard regression model. ^ Confidence interval of the estimated HR.

## Data Availability

Relevant data supporting the findings of this study are available within the article and supplementary information and are available from the authors upon reasonable request. Ovarian serous cystadenocarcinoma datasets publicly available on cbioportal (https://www.cbioportal.org/, accessed on 5 August 2020) were interrogated: TCGA, Firehouse legacy.

## Supplementary Materials

The following are available online at https://www.mdpi.com/article/10.3390/cancers14041039/s1, Figure S1: pGSN expression and infiltrated M2 macrophages in OVCA tissues, Figure S2: Epithelial pGSN expression, M1/M2 density and survival in OVCA histologic subtypes, Figure S3: Higher infiltration of M1 but not M2 macrophages is associated with improved survival, Figure S4: Epithelial pGSN expression is significantly correlated with M1/M2 macrophage ratio, Figure S5: pGSN positively correlates with M1 macrophage genes in HGSC, Figure S6: M1 macrophage differentiation and polarization, Figure S7: pGSN knock-down and over-expression confirmation in sEVs and rhpGSN-induced apoptosis in M1 macrophages, Figure S8: NAC suppresses ROS production induced by NO in chemoresistant OVCA cells, Figure S9: Epithelial pGSN expression increases with increased tumor stage and suboptimal residual disease. Table S1: Characteristics of patients, Table S2: Information on antibodies and reagents, Table S3: Information on OVCA cell lines, Table S4: pGSN expression and TAMs infiltration in OVCA tissue compartments.
