# Supplementary material for: Plasma Gelsolin Confers Chemoresistance in Ovarian Cancer by Resetting the Relative Abundance and Function of Macrophage Subtypes
Source: Cancers (Basel). 2022 Feb 18;14(4):1039. doi: 10.3390/cancers14041039 (PMC8870487; doi:10.3390/cancers14041039)
Supplement: Supplementary file 1 [file cancers-14-01039-s001.zip › cancers-1523799-supplementary.pdf]

Supplementary figures

Supplementary Figure S1

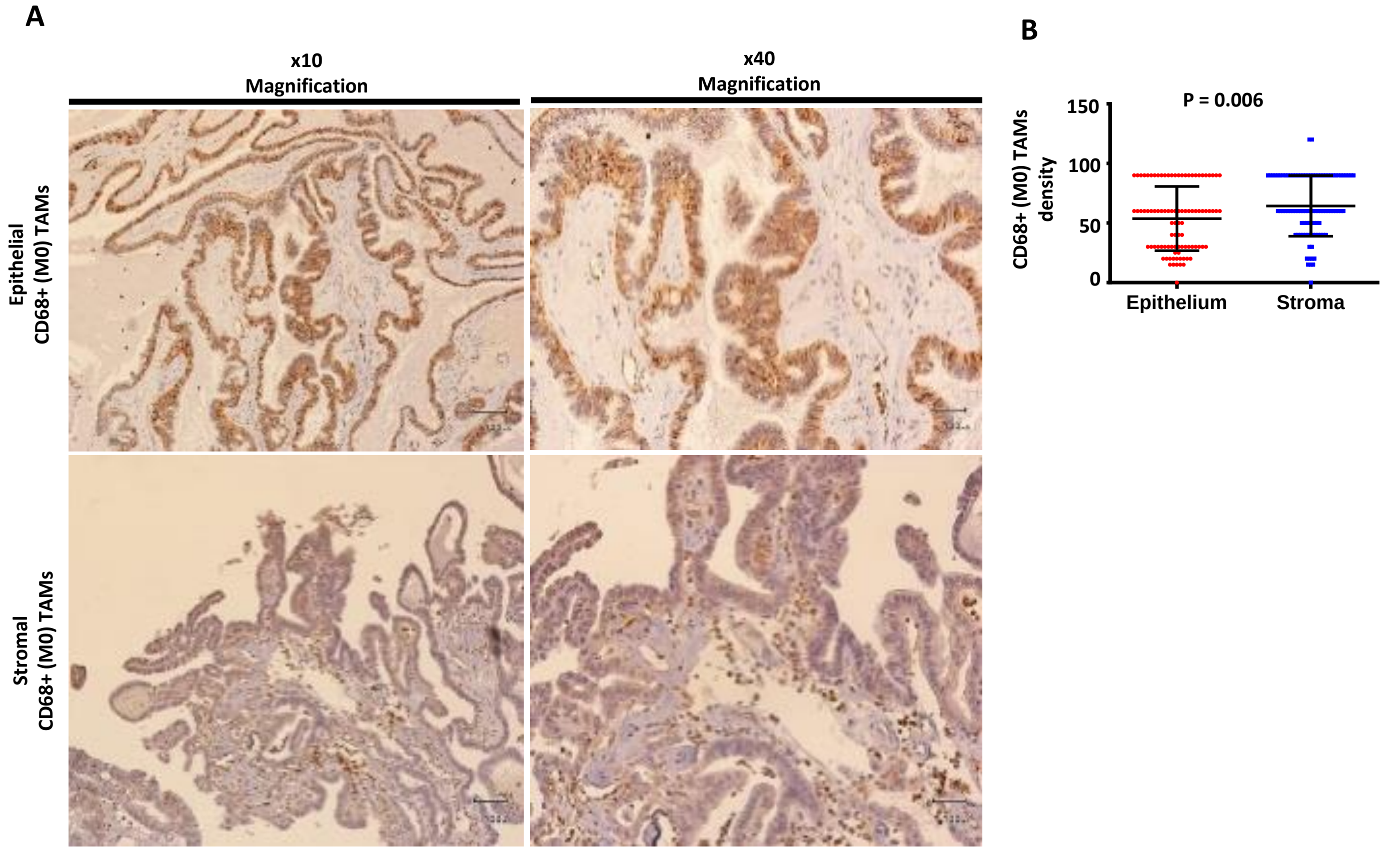

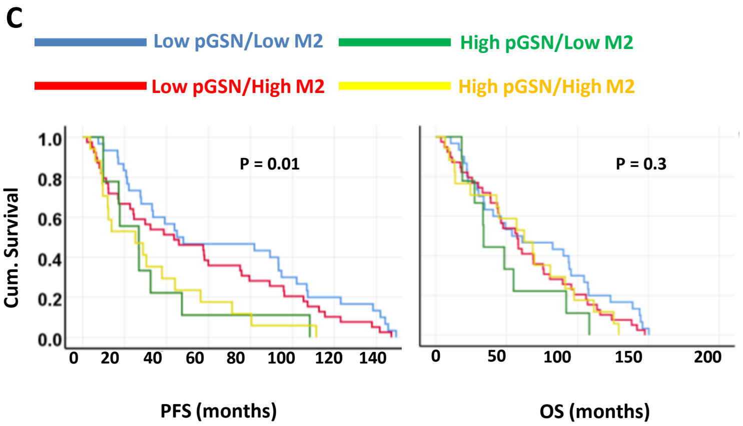

Mean survival	PFS (months)	OS (months)
**Low pGSN Low M2**	**68.1**	**71.2**
Low pGSN High M2	54.3	62.4
**High pGSN Low M2**	**32.9**	**48.5**
**High pGSN High M2**	**33.0**	**61.8**

Sup. Fig. S1. pGSN expression and infiltrated M2 macrophages in OVCA tissues. (A) 94 OVCA tissues were immunostained with anti-pGSN, anti-CD68 (M0 macrophage) antibodies in the epithelial and stromal compartments. **(B)** Tissue CD68+ macrophages were quantified, compared between epithelial (n=94) and stromal (n=94) regions and presented as scatter plots (mean ± SD). P-values were calculated by independent sample t-test. Scale bar is 50 µm. **(C)** pGSN expression (cut-off = 6) was assessed together with infiltrated M2 (cut-off = 60) macrophages in the epithelial region and then correlated with PFS and OS. Kaplan Meier survival curves with cut-off values and log rank test were used to compare the survival distributions between the groups.

Supplementary Figure S2

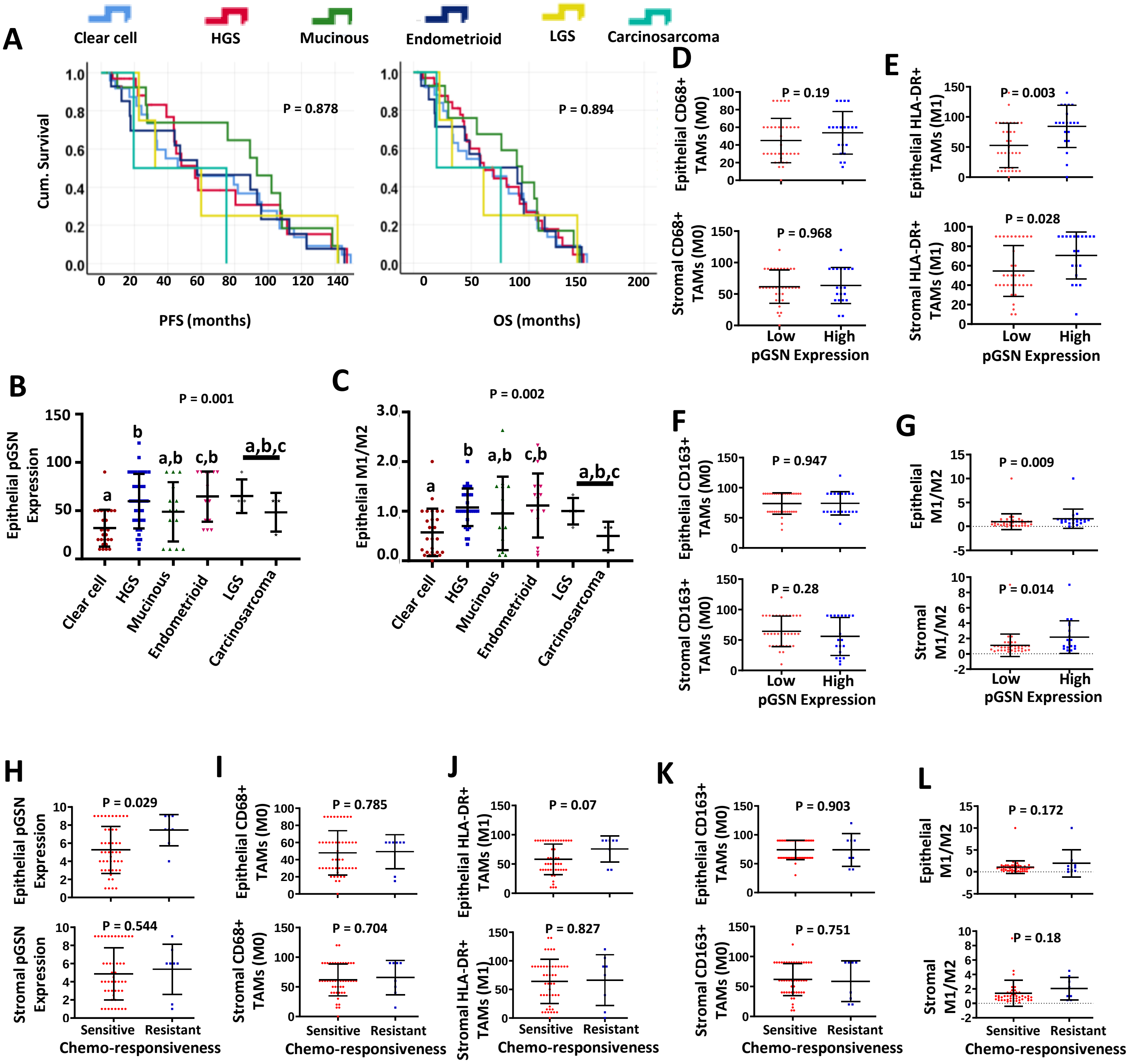

Sup. Fig. S2. Epithelial pGSN expression, M1/M2 density and survival in OVCA histologic subtypes. (A) Patients were stratified using their histologic subtypes (Clear cell; n=26, HGS; n=33, Mucinous; n=14, Endometrioid; n=14, LGS; n=4, Carcinosarcoma; n=3) and correlated with PFS and OS. Kaplan Meier survival curves and log rank test were used to compare the survival distributions between the groups. **(B)** Epithelial pGSN expression was compared between patients with Clear cell; n=26, HGS; n=33, Mucinous; n=14, Endometrioid; n=14, LGS; n=4 and Carcinosarcoma; n=3. **(C)** Epithelial M1/M2 was calculated and compared between patients with Clear cell; n=26, HGS; n=33, Mucinous; n=14, Endometrioid; n=14, LGS; n=4 and Carcinosarcoma; n=3. n = number of patients in each group. OVCA patients at stage 1 were stratified into two groups depending on their level of pGSN expression (low vs high). (D) M0, (E) M1, (F) M2 and (G) M1/M2 quantities in the epithelium and stroma were compared and represented as scatter plots (mean ± SD). P-values were calculated by independent sample t-test. Patients were also grouped into chemoresistant (PFI ≤ 12 months) and chemosensitive (PFI > 12 months) groups. Epithelial and stromal expressions of (H) pGSN, (I) M0, (J) M1, (K) M2 and (L) M1/M2 were quantitated, compared and represented as scatter plots (mean ± SD). P-values were calculated by two sided non-parametric Mann-Whitney test. The groups were compared using Kruskal-Wallis test (with Dunn's multiple comparison test). [**B**, (a; **p<0.01 vs b), (a; **p<0.01 vs c); **C**, (a; **p<0.01 vs b), (a; *p<0.05 vs c)].

Supplementary Figure S3

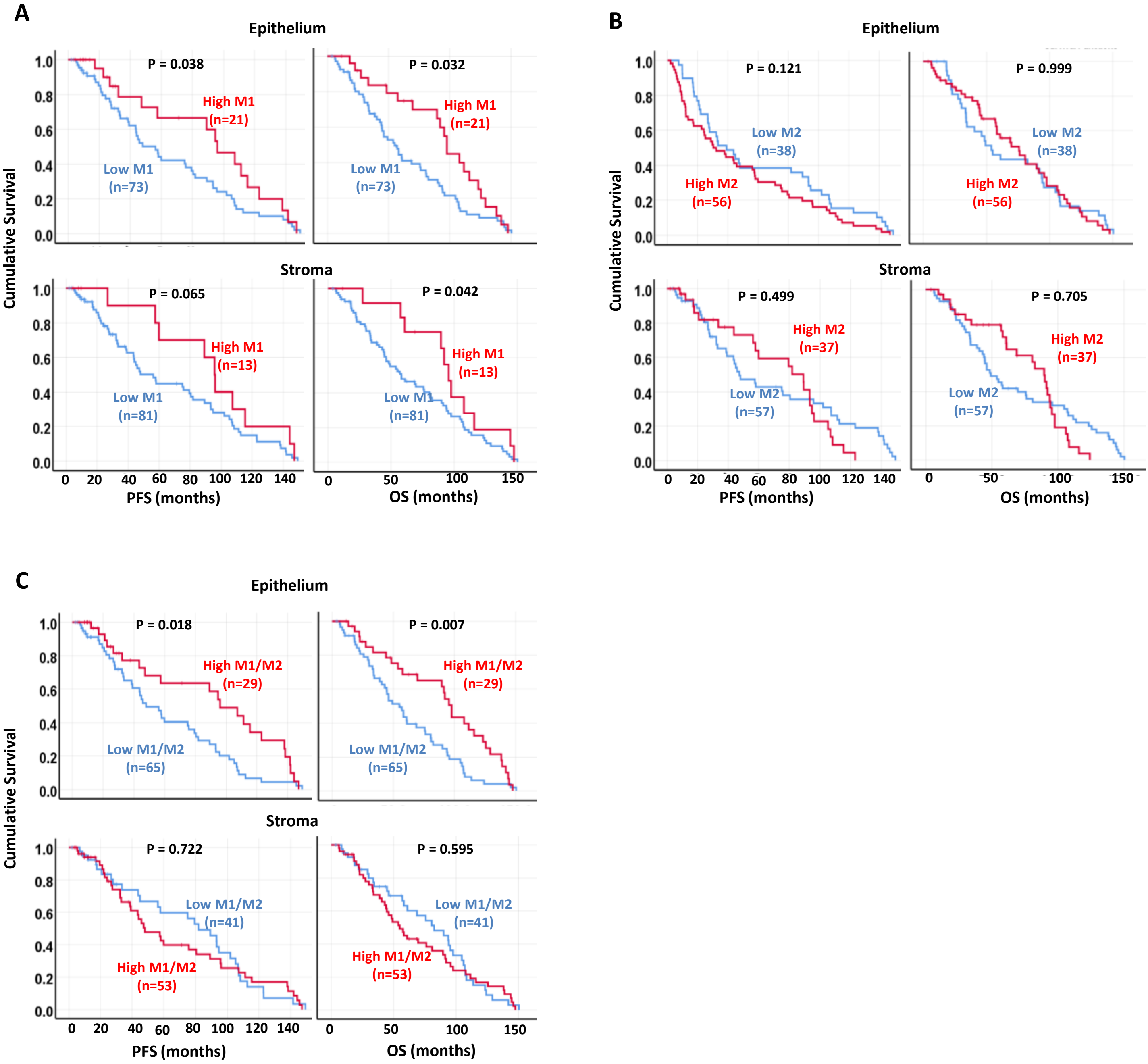

Sup. Fig. S3. Higher infiltration of M1 but not M2 macrophages is associated with improved survival. (A) HLA-DR+ M1 (cut-off = 60), **(B)** CD163+ M2 (cut-off = 60) and **(C)** M1/M2 infiltrated macrophages (cut-off = 1) in the epithelial and stromal regions were correlated with PFS and OS. Kaplan Meier survival curves with cut-off values and log rank test were used to compare the survival distributions between the groups. n = number of patients in each group.

Supplementary Figure S4

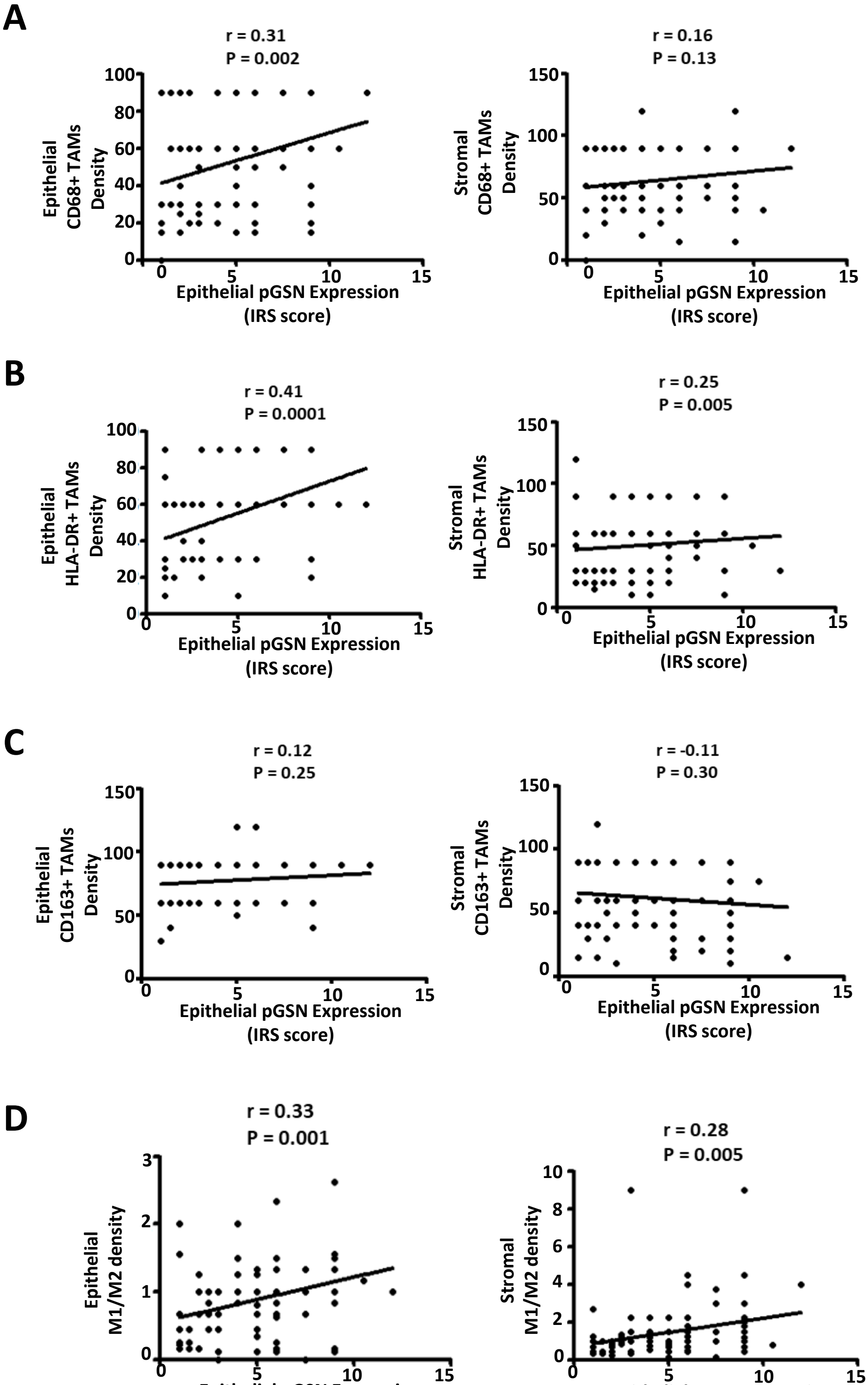

Sup. Fig. S4. Epithelial pGSN expression is significantly correlated with M1/M2 macrophage ratio. Epithelial pGSN expression was correlated with epithelial and stromal densities of **(A)** M0, **(B)** M1, **(C)** M2 macrophages and **(D)** M1/M2 macrophage ratio. Pearson's analysis was used to examine the correlation between epithelial pGSN and TAMs.

Supplementary Figure S5

A

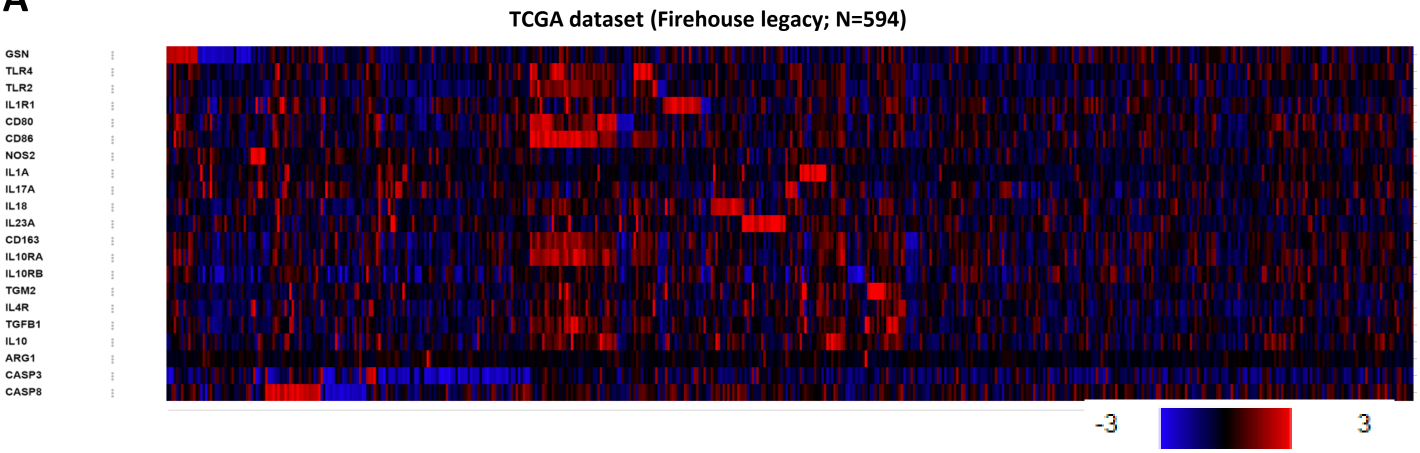

B

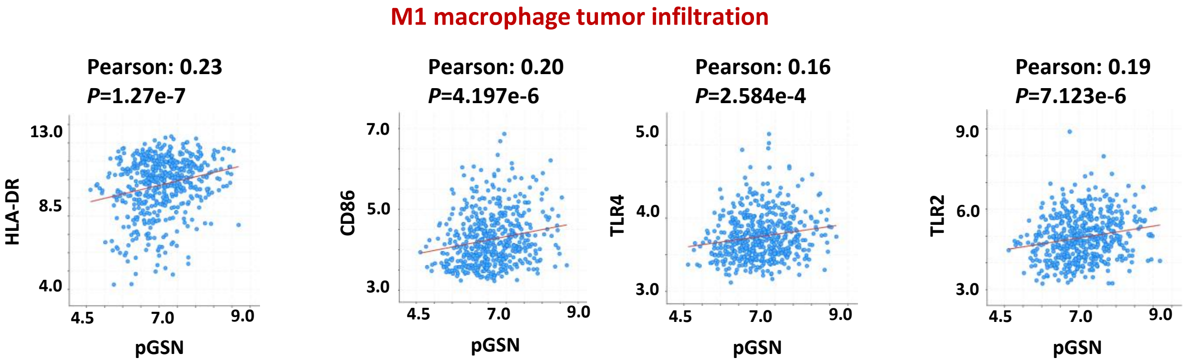

Sup. Fig. S5. pGSN positively correlates with M1 macrophage genes in HGSC (A) Heat map and **(B)** Pearson's analysis of the association between M1 macrophage genes and pGSN mRNA expression. Data was interrogated from Firehouse legacy datasets publicly available on cbioportal.

Supplementary Figure S6

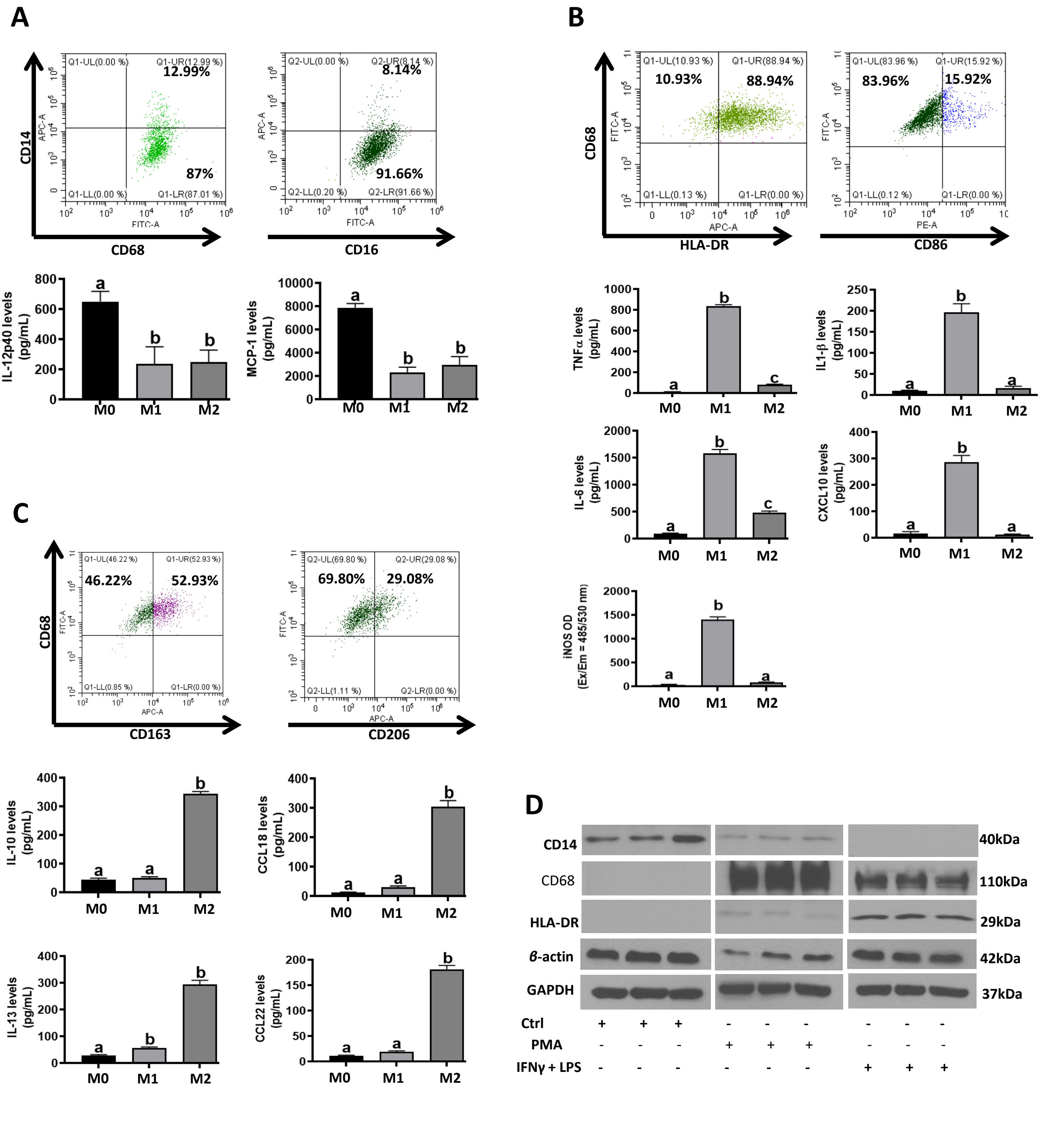

Sup. Fig. S6. M1 macrophage differentiation and polarization. (A) THP-1 monocytes were differentiated into M0 macrophages by PMA treatment (150 nM; 24 h). M0 macrophages were polarized to M1 macrophages with IFNγ (20ng/ml) + LPS (10pg/ml) for 24 h and M2 macrophages by IL-4 (20ng/ml + IL-13 (20ng/ml) for 72 h. Differentiation and polarization were confirmed by **(A-C)** flow cytometry, ELISA (cytokines), fluorometric assay (iNOS) and **(D)** Western blot (CD14, CD68, HLA-DR, beta actin and GAPDH).

Supplementary Figure S7

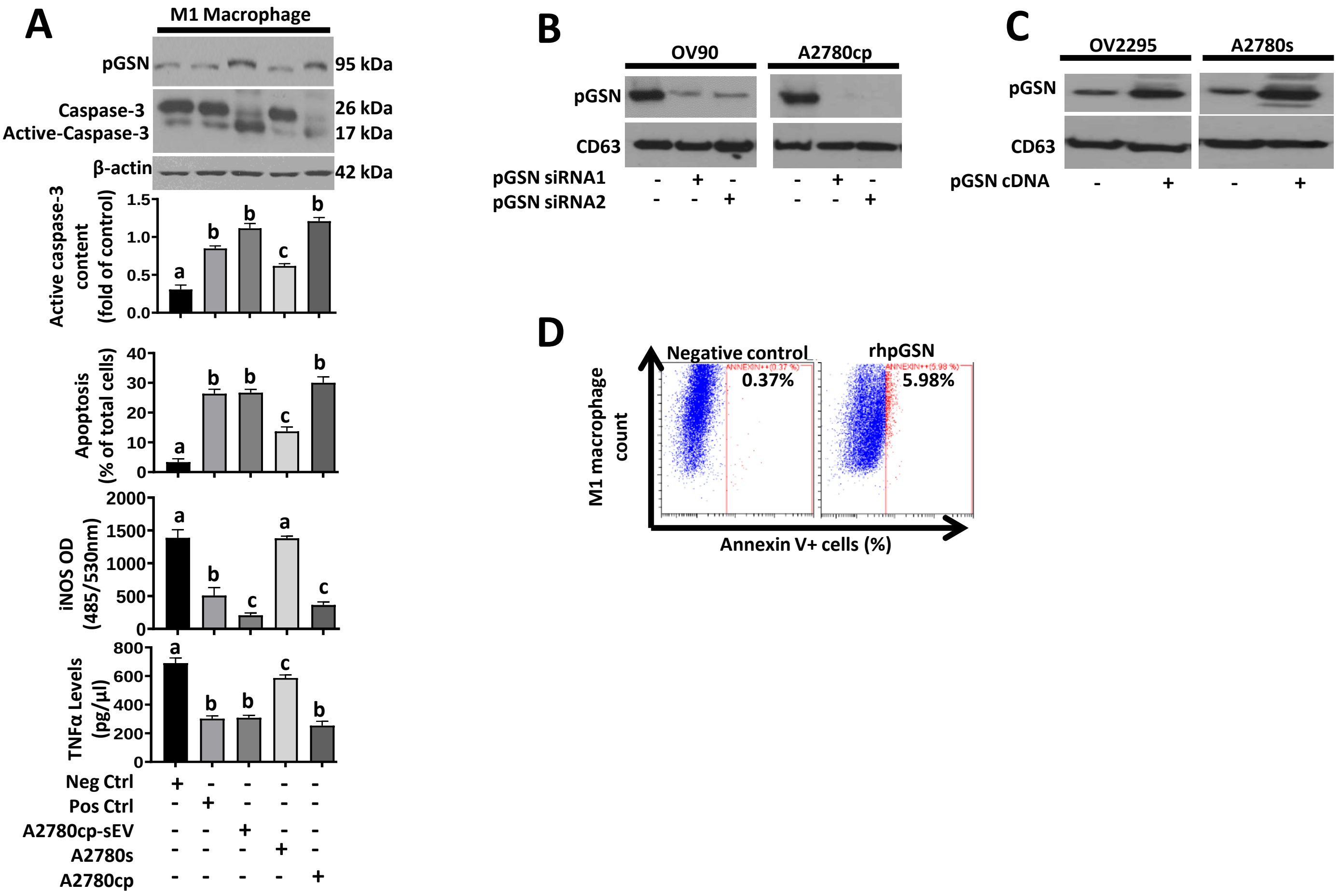

Sup. Fig. S7. pGSN knockdown and overexpression confirmation in sEVs and rhpGSN-induced apoptosis in M1 macrophages. (A) M1 macrophages were co-cultured with serum-free media (negative control; 48 h), etoposide (0.5 µM; 48 h), A2780cp-derived sEV (40 µg/400,000 cells; 48 h), A2780s (48 h) and A2780cp (48 h). Pro-caspase-3, activated caspase-3, pGSN and beta-actin contents were assessed by Western blot (M1 macrophage lysates). **(B)** Chemoresistant OVCA cells (OV90 and A2780cp) were treated with pGSN siRNA1 and 2 (50 nM; 24 h). **(C)** Chemosensitive OVCA cells (OV2295 and A2780s) were treated with pGSN cDNA (2 µg; 24 h). Conditioned media from treated cells were collected and sEVs isolated. pGSN and sEV marker (CD63) contents were assessed by Western blotting. **(D)** M1 macrophages were treated with rhpGSN (10 µM; 24 h). DMSO was used as control. Apoptosis was analysed by counting Annexin V+ M1 macrophages using flow cytometry. Results are expressed as means ± SD from three independent replicate experiments. [**A**, (a; ***p<0.001 vs b and c)]

Supplementary Figure S8

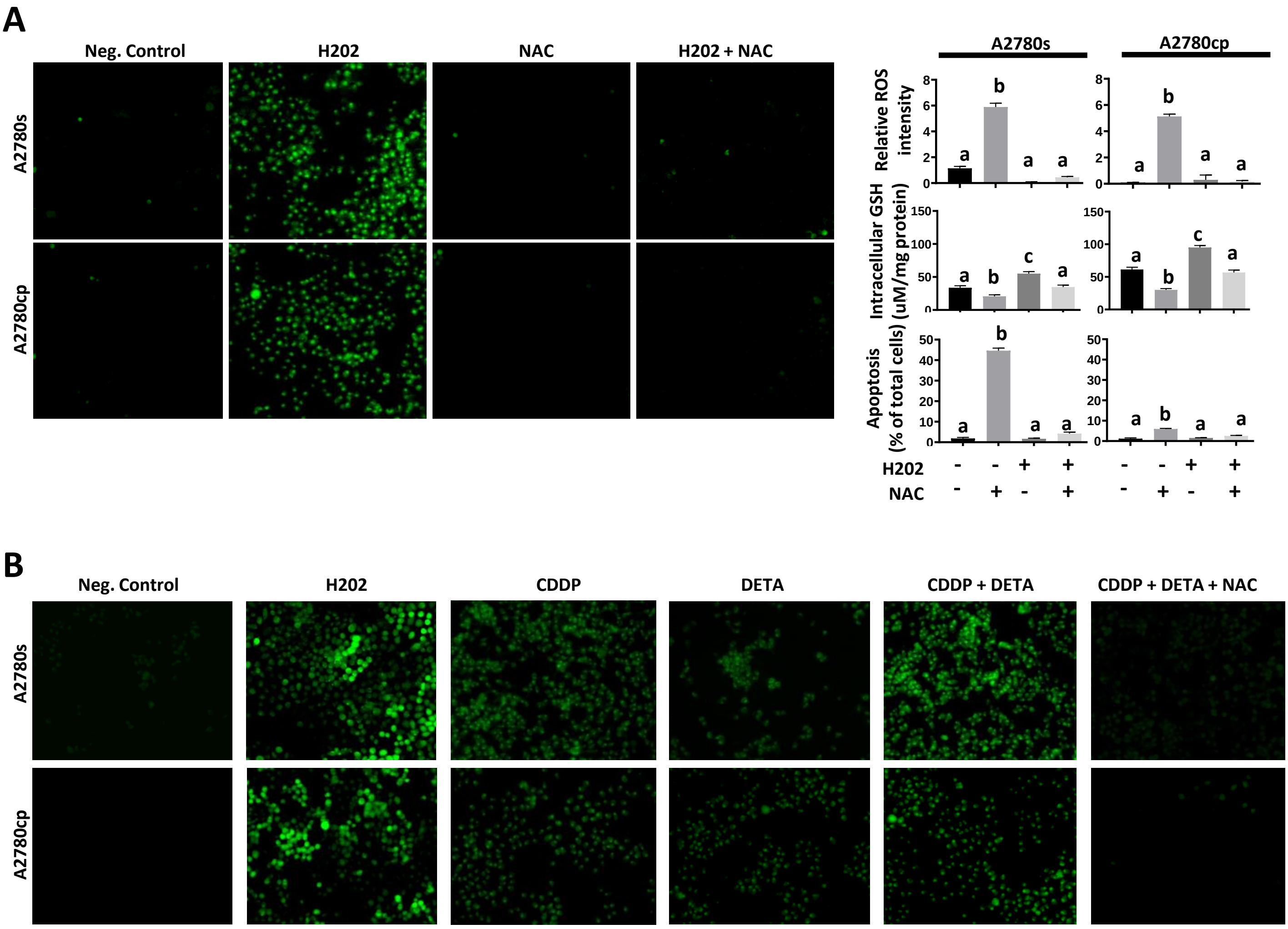

Sup. Fig. S8. NAC suppresses ROS production induced by NO in chemoresistant OVCA cells. (A) A2780s and A2780cp cells were treaed with H_20_2 (positive control; 2 mM), N-acetylcysteine [GSH precursor (NAC; 200 µM)] and H_20_2 (2 mM)+N-acetylcysteine [GSH precursor (NAC; 200 µM)] for 6-12 h. **(B)** A2780s and A2780cp cells were treated with H_20_2 (positive control; 2 mM), CDDP (10 µM), DETA NONOate (NO donor, 200 µM), CDDP+DETA NONOate and CDDP (10 µM)+DETA NONOate (200 µM)+N-acetylcysteine [GSH precursor (NAC; 200 µM)] for 6 – 12 h. Intracellular GSH was measured by colorimetric assay and ROS determined by 2',7'-Dichlorofluorescein diacetate assay. Apoptosis morphologically determined by Hoechst 33258 DNA staining. Results are expressed as means ± SD from three independent replicate experiments. [**A**, (a; ***p<0.001 vs b and c)]. Scale bar is 100 µm.

Supplementary Figure S9

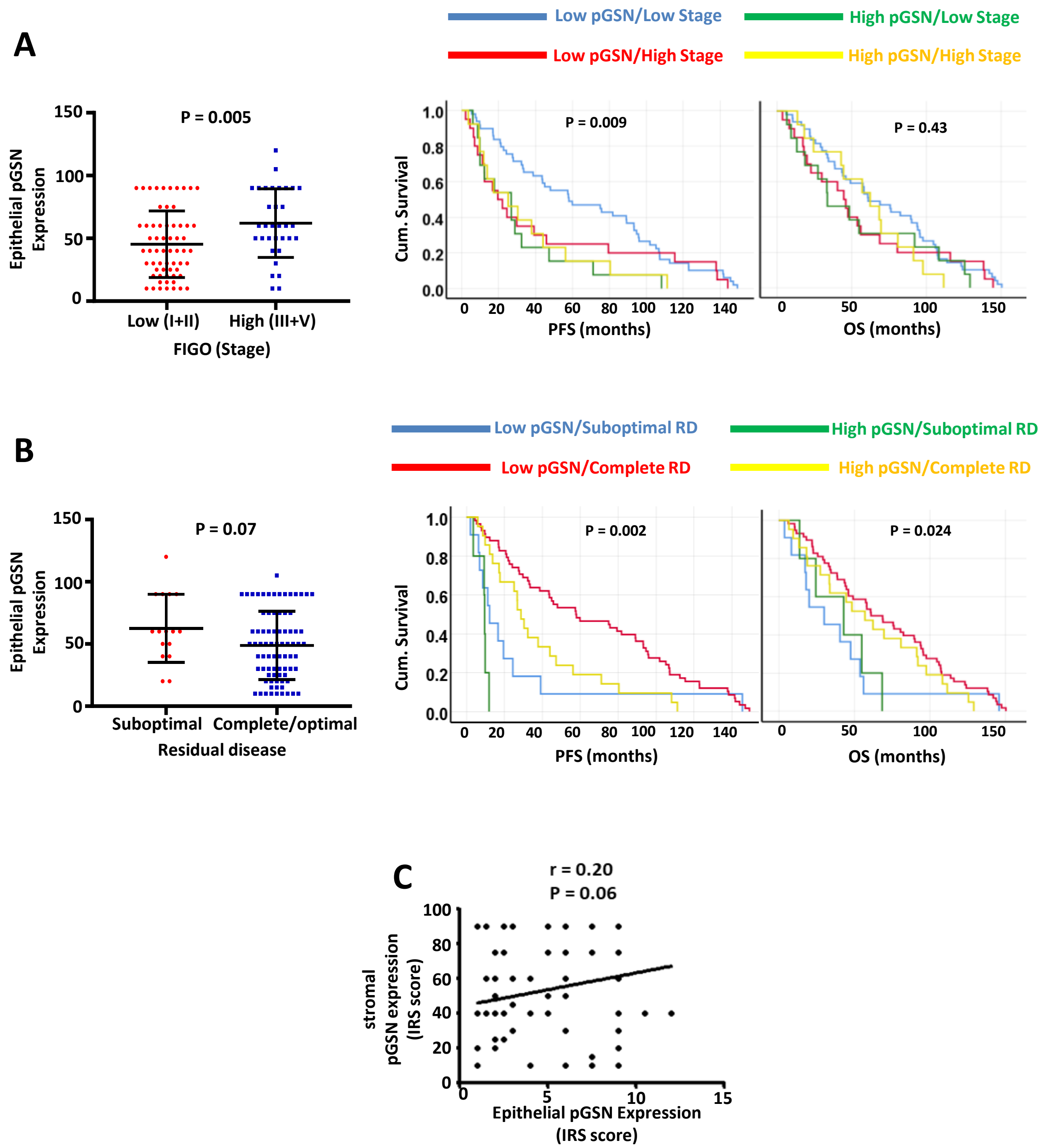

Sup. Fig. S9. Epithelial pGSN expression increases with increased tumor stage and suboptimal residual disease. (A) Epithelial pGSN expression was compared between patients with low (n=62) and high (n=32) stage. Patients were stratified using epithelial pGSN and tumor stage . **(B)** Epithelial pGSN expression was compared between patients with complete/optimal (n=79) and suboptimal (n=15) RD. Patients were stratified using epithelial pGSN and residual disease. pGSN (cut-off = 6) in combination with tumor stage and residual disease were correlated with PFS and OS. Kaplan Meier survival curves with cutoff values and log rank test were used to compare the survival distributions between the groups. **(C)** Epithelial pGSN was correlated with stromal pGSN expression using Pearson's analysis. n = number of patients in each group. Groups in **(A)** and **(B)** were compared using independent sample t test.

Supplementary Figure S10

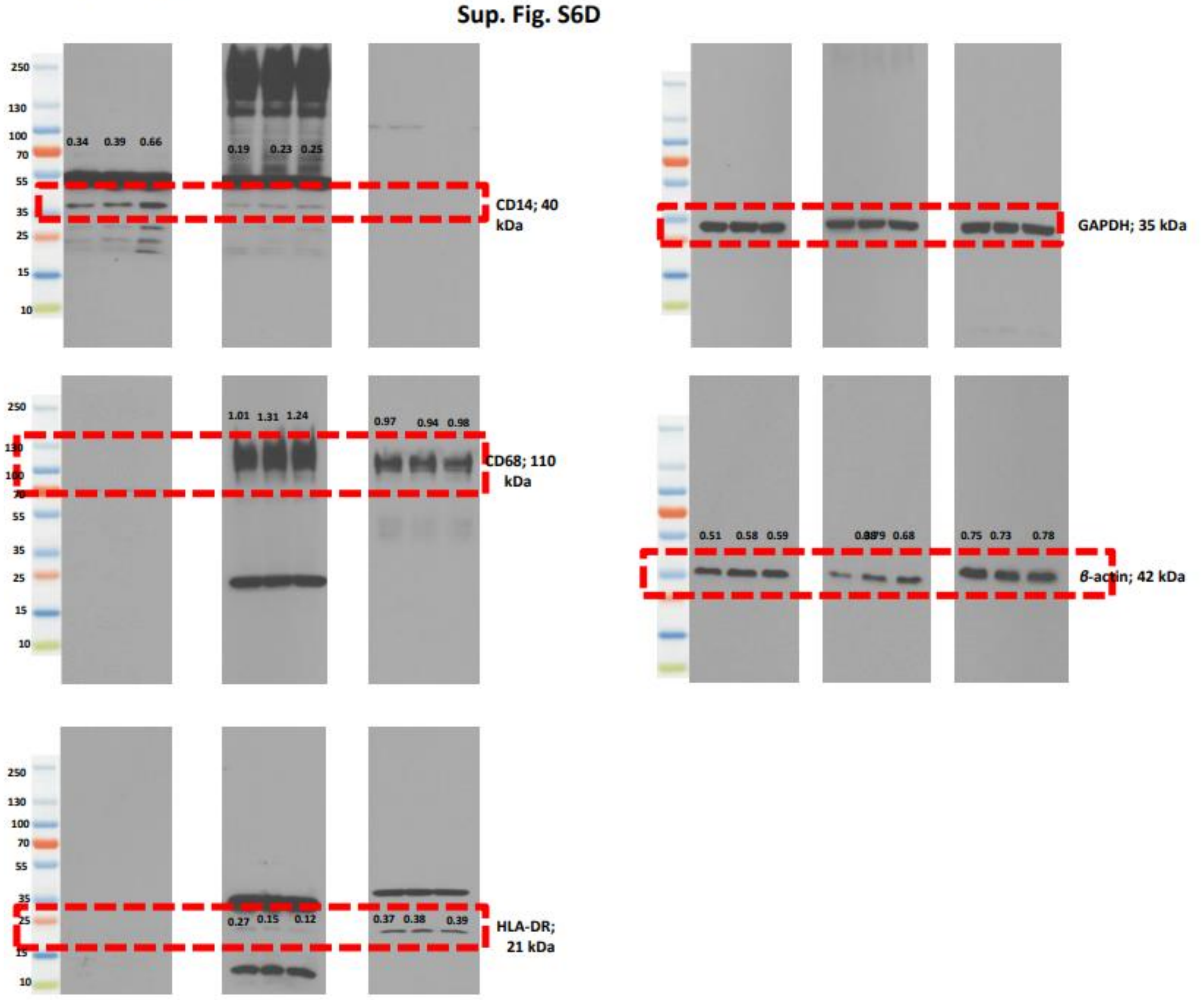

Sup. Fig. S10. Western blot data. Proteins were probed with their respective primary antibodies (CD14, CD68, HLA-DR, actin, GAPDH) and then with the appropriate horseradish peroxidase (HRP)-conjugated secondary antibodies. Peroxidase activity was visualized and developed by using a chemiluminescent kit (refer to materials and methods) onto an x-ray film. The films were cut out and scanned using a RICOH IM C4500.

Supplementary Figure S11

Sup. Fig. S11

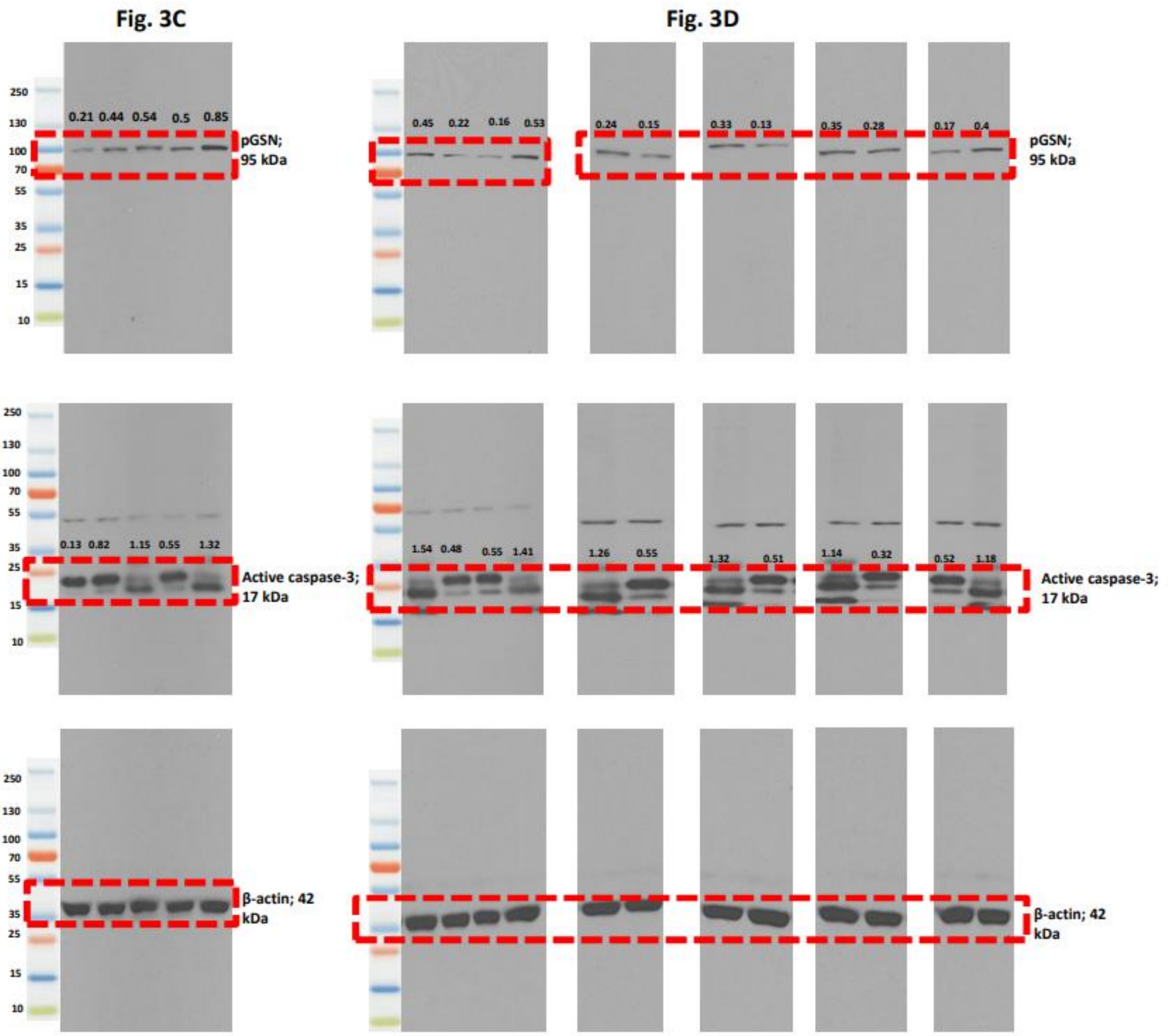

Sup. Fig. S11. Western blot data. Proteins were probed with their respective primary antibodies (pGSN, active caspae-3, actin) and then with the appropriate horseradish peroxidase (HRP)-conjugated secondary antibodies. Peroxidase activity was visualized and developed by using a chemiluminescent kit (refer to materials and methods) onto an x-ray film. The films were cut out and scanned using a RICOH IM C4500.

Supplementary Figure S12

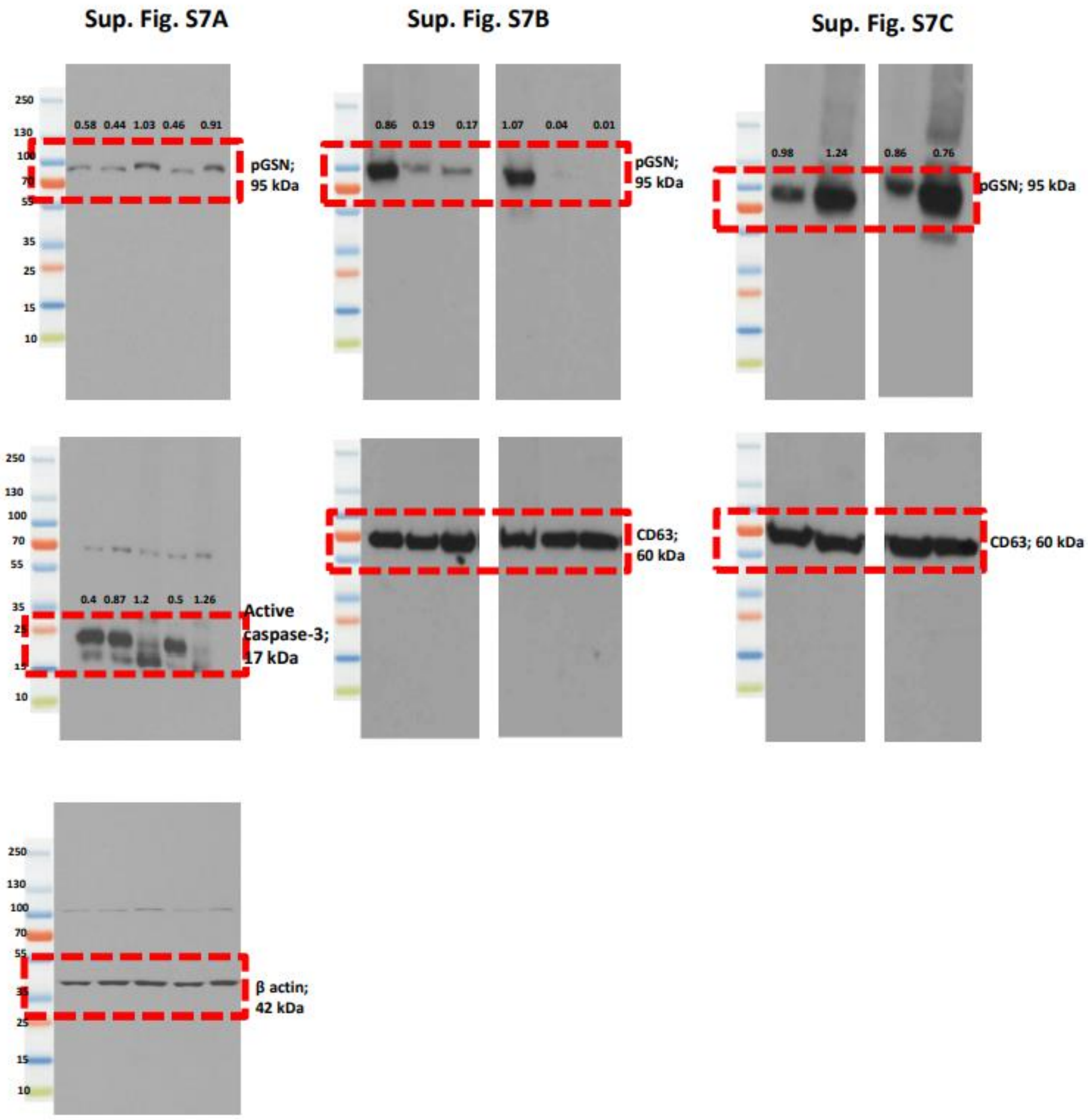

Sup. Fig. S12. Western blot data. Proteins were probed with their respective primary antibodies (pGSN, CD63, active caspase-3 and actin) and then with the appropriate horseradish peroxidase (HRP)-conjugated secondary antibodies. Peroxidase activity was visualized and developed by using a chemiluminescent kit (refer to materials and methods) onto an x-ray film. The films were cut out and scanned using a RICOH IM C4500.

Supplementary Figure S13

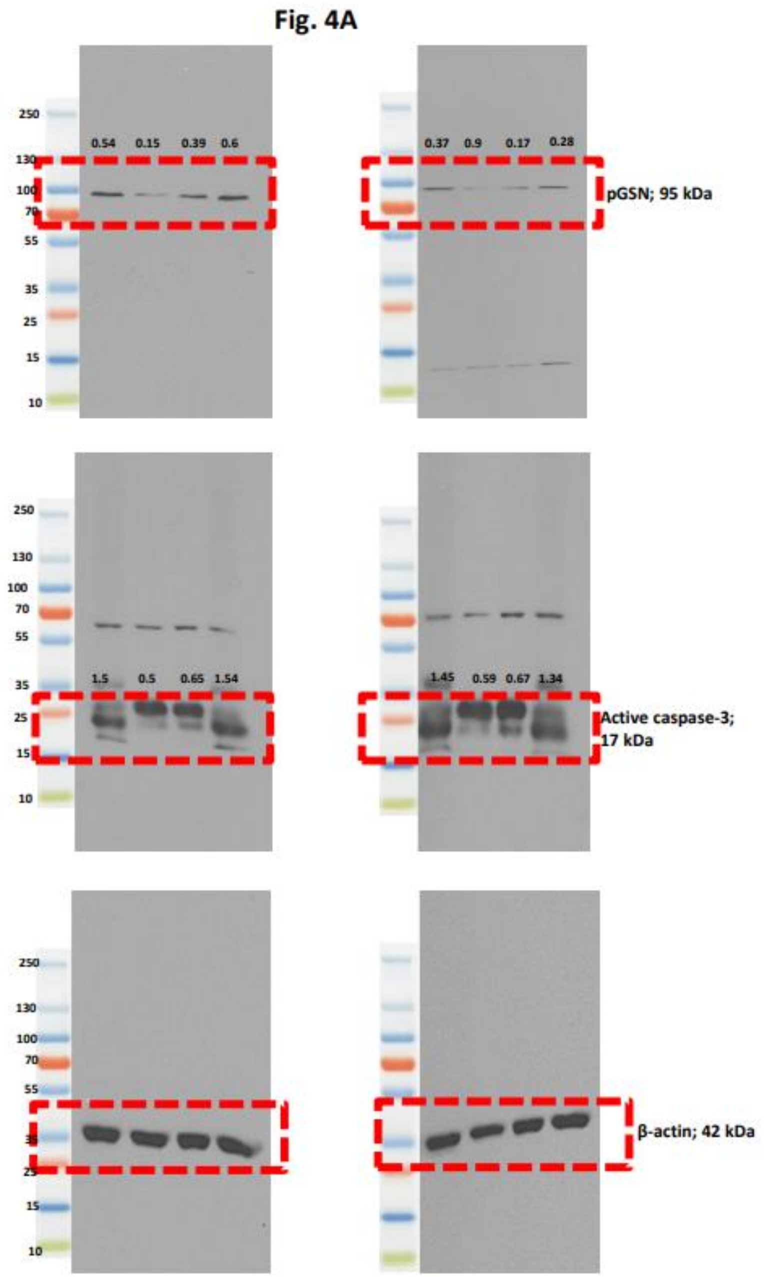

Sup. Fig. S13. Western blot data. Proteins were probed with their respective primary antibodies (pGSN, active caspase-3 and actin) and then with the appropriate horseradish peroxidase (HRP)-conjugated secondary antibodies. Peroxidase activity was visualized and developed by using a chemiluminescent kit (refer to materials and methods) onto an x-ray film. The films were cut out and scanned using a RICOH IM C4500.

Plasma gelsolin confers chemoresistance in ovarian cancer by resetting the relative abundance and function of macrophage subtypes

Meshach Asare-Werehene[1,2,3], Hideaki Tsuyoshi[4], Huilin Zhang[3,5], Reza Salehi[1,2,3,8], Chia-Yu Chang[6], Euridice Carmona[7], Clifford L. Librach[8,9], Anne-Marie Mes-Masson[7], Chia-Ching Chang[6], Dylan Burger[2,3], *Yoshio Yoshida[4], *Benjamin K. Tsang[1,2,3]

Running Title: pGSN overexpression suppresses M1 macrophage functions.

Supplementary Table S1. Characteristics of patients

Variable	Number of Patients
Age (Range; 29 – 87 years)	
≤56	51
>56	43
Stage (FIGO)	
1	55
2	9
3	23
4	7
Stage (FIGO)	
≤2	64
>2	30
Histological Subtypes	
Clear cell	26
HGS	33
Mucinous	14
Endometrioid	14
LGS	4
Carcinosarcoma	3
Residual disease (RD)	
≤1 cm	79
>1 cm	15
Progression-free survival (PFS)	
Recurrent	26
Non-recurrent	68
Median PFS	33.5 months
Overall Survival (OS)	
Deceased	14
Alive	80
Median OS	56.2 months

Inclusion Criteria	Exclusion Criteria
Histologically confirmed epithelial ovarian cancer, fallopian tube cancer, or peritoneal cancer.	Other malignancy within the last 5 years except: adequately treated non-melanoma skin cancer curatively treated in situ cancer of the cervix, ductal carcinoma in situ.
Female Patient must be ≥20 years of age.	Patients judged ineligible as subjects of this study by the investigator.
Signed informed consent and ability to comply with treatment and follow-up.	
ECOG performance status 0-2.	

HGSC, high grade serous carcinoma; RD, residual disease; FIGO, International Federation of Gynecology and Obstetrics

Supplementary Table S2. Information on antibodies and Reagents

Application	Target	Primary Antibody/reagent	Company	Catalog #	Dilution	Secondary Antibody	Conjugate	Company	Catalog #	Dilution
**WB**	pGSN	Anti-pGSN Goat polyclonal	Antibodies online(Atlanta, USA)	ABIN1019662	1:1000	Dnk polyclonal to Goat IgG	HRP	Abcam (Toronto, Canada)	Ab97110	1:2000
**WB**	CD63	Anti-CD63 mouse monoclonal	Abcam (Toronto, Canada)	Ab193349	1:1000	Goat Anti-mouse IgG (H+L)	HRP	Bio-Rad (Mississauga, Canada)	170-6516	1:2000
**WB**	β-actin	Anti-actin mouse monoclonal	Abcam (Toronto, Canada)	ab8226	1:1000	Goat Anti-mouse IgG (H+L)	HRP	Bio-Rad (Mississauga, Canada)	170-6516	1:2000
**IHC**	pGSN	Anti-pGSN mouse polyclonal	Antibodies online(Atlanta, USA)	ABIN659182	1:1000	MAX-PO (MULTI)				
**IHC**	HLA-DR	Anti-HLA-DR mouse monoclonal	Abcam (Canada)	Ab20181	1:1000	MAX-PO (MULTI)				
**IHC**	CD68	Anti-CD68 rabbit polyclonal	Abcam (Canada)	Ab125212	1:50	MAX-PO (MULTI)				
**IHC**	CD163	Anti-CD163 rabbit polyclonal	Abcam (Canada)	Ab87099	1:400	MAX-PO (MULTI)				
**WB**	CD14	Anti-CD14 rabbit monoclonal	Abcam (Canada)	Ab183322	1:1000	Goat Anti-mouse IgG (H+L)	HRP	Bio-Rad (Mississauga, Canada)	170-6516	1:2000
**WB**	HLA-DR	Anti-HLA-DR mouse monoclonal	Abcam (Canada)	Ab20181	1:1000	Goat Anti-mouse IgG (H+L)	HRP	Bio-Rad (Mississauga, Canada)	170-6516	1:2000
**WB**	CD68	Anti-CD68 rabbit polyclonal	Abcam (Canada)	Ab125212	1:1000	Goat Anti-rabbit IgG (H+L)	HRP	Bio-Rad (Mississauga, Canada)	170-6515	1:2000
**WB**	CD163	Anti-CD163 rabbit polyclonal	Abcam (Canada)	Ab87099	1:1000	Goat Anti-rabbit IgG (H+L)	HRP	Bio-Rad (Mississauga, Canada)	170-6515	1:2000
**WB**	Activated caspase-3	Anti-cleaved caspase-3 rabbit polyclonal	Cell signaling Technology, MA, USA	9661	1:1000	Goat Anti-rabbit IgG (H+L)	HRP	Bio-Rad (Mississauga, Canada)	170-6515	1:2000
**iNOS detection**	iNOS		Abcam (Canada)	Ab211085						
**TNF alpha ELISA kit**	TNF alpha		Abcam (Canada)	Ab46087						
**Caspase-3 activation detection**	Caspase-3		Thermofisher Scientific (Canada)	C10723						
**Annexin V-FITC**	Annexin V		Fisher Scientific (Canada)	50-930-1						
		PMA	Sigma (Canada)	P8139-1MG						
		LPS	Sigma (Canada)	L2630-10MG						
		hrIFN gamma	Stemcell Technologies (Canada)	78020						
		Cisplatin	Sigma (Canada)	P4394-100MG						
		Etoposide	Sigma (Canada)	E1383-25MG						
**Flow cytometry**	CD14	APC mouse anti-human clone M5E2	BD	555399						
**Flow cytometry**	CD68	FITC mouse anti-human clone Y1/82A	BD	562117						
**Flow cytometry**	HLA-DR	APC mouse anti-human clone G46-6	BD	559866						
**Flow cytometry**	CD86	PE mouse anti-human clone 2331 (FUN-1)	BD	557344						
**Flow cytometry**	CD163	Alexa Flour 647 mouse anti-human clone GHI/61	BD	562669						
**Flow cytometry**	CD206	PE mouse anti-human clone 19.2	BD	555954						
**Flow cytometry**	CD16	FITC mouse anti-human clone 3G8 (RUO)	BD	555406						
		N-acetyl-L-Cysteine (NAC)	Sigma Millipore	A9165-25G						
		1400W dihydrochloride	R&D systems	1415/10						
		Hydrogen peroxide	Sigma Millipore	386790						
		DETA-NONOate	Abcam	Ab144627						

		CCK-8	Sigma Millipore	96992						
		hrIL4	Stemcell Technologies	78045.1						
		hrIL13	Stemcell Technologies	78029.1						
		hrCCL19	Peprotech	300-29B						
		2′,7′-Dichlorofluorescein diacetate	Sigma Millipore	D6883						

Supplementary Table S3. Information on OVCA cell lines

Cell line	Tumor origin	TP53 status	Other	Chemosensitivity
**A2780s**	Ovarian endometrioid adenocarcinoma	Wild type	PTEN/ARID1A	Sensitive
**A2780cp**	Ovarian endometrioid adenocarcinoma	Mutant V127F, R260S	PTEN/ARID1A	Resistant
**OV2295**	High grade serous ovarian cancer	Mutant IIe195Thr	Not investigated	Sensitive
**OV90**	High grade serous ovarian cancer	Mutant Ser215Arg	Not investigated	Resistant
**TOV3041G**	High grade serous ovarian cancer	Wild type	Not investigated	Sensitive

Information on OVCA cell lines: The characterization of these cell lines have been verified in previous literature (Anglesio et al., 2013; Leroy et al., 2014; Provencher et al., 2000; Letourneau et al., 2012; Fleury et al., 2015).

Supplementary Table S4. pGSN expression and TAMs infiltration in OVCA tissue compartments

Tissue Marker Expression	Number of Patients
**Epithelial pGSN (IRS)**	
≤6 (Low)	68
>6 (High)	26
**Stromal pGSN (IRS)**	
≤6 (Low)	62
>6 (High)	32
**Epithelial CD68+ (M0)**	
≤6 (Low)	68
>6 (High)	26
**Stromal CD68+ (M0)**	
≤6 (Low)	57
>6 (High)	37
**Epithelial HLA-DR+ (M1)**	
≤6 (Low)	73
>6 (High)	21
**Stromal HLA-DR+ (M1)**	
≤6 (Low)	81
>6 (High)	13
**Epithelial CD163+ (M2)**	
≤6 (Low)	38
>6 (High)	56
**Stromal CD163+ (M2)**	
≤6 (Low)	57
>6 (High)	37
**Epithelial M1/M2 Density**	
≤1 (Low)	65
>1 (High)	29
**Stromal M1/M2 Density**	
≤6 (Low)	53
>6 (High)	41

IRS, immunoreactive score; pGSN, plasma gelsolin
